# Tardigrade workbench: comparing stress-related proteins, sequence-similar and functional protein clusters as well as RNA elements in tardigrades

**DOI:** 10.1186/1471-2164-10-469

**Published:** 2009-10-12

**Authors:** Frank Förster, Chunguang Liang, Alexander Shkumatov, Daniela Beisser, Julia C Engelmann, Martina Schnölzer, Marcus Frohme, Tobias Müller, Ralph O Schill, Thomas Dandekar

**Affiliations:** 1 Dept of Bioinformatics, Biocenter University of Würzburg, 97074 Würzburg, Germany; 2 EMBL, Hamburg Outstation, Notkestrasse 85, 22603 Hamburg, Germany; 3 Functional Proteome Analysis, German Cancer Research Center, Im Neuenheimer Feld 580, 69120 Heidelberg, Germany; 4 University of Applied Sciences, Bahnhofstraße 1, 15745 Wildau, Germany; 5 Dept of Zoology, Institute for Biology, Universität Stuttgart, 70569 Stuttgart, Germany

## Abstract

**Background:**

Tardigrades represent an animal phylum with extraordinary resistance to environmental stress.

**Results:**

To gain insights into their stress-specific adaptation potential, major clusters of related and similar proteins are identified, as well as specific functional clusters delineated comparing all tardigrades and individual species (*Milnesium tardigradum*, *Hypsibius dujardini*, *Echiniscus testudo*, *Tulinus stephaniae*, *Richtersius coronifer*) and functional elements in tardigrade mRNAs are analysed. We find that 39.3% of the total sequences clustered in 58 clusters of more than 20 proteins. Among these are ten tardigrade specific as well as a number of stress-specific protein clusters. Tardigrade-specific functional adaptations include strong protein, DNA- and redox protection, maintenance and protein recycling. Specific regulatory elements regulate tardigrade mRNA stability such as lox P DICE elements whereas 14 other RNA elements of higher eukaryotes are not found. Further features of tardigrade specific adaption are rapidly identified by sequence and/or pattern search on the web-tool tardigrade analyzer http://waterbear.bioapps.biozentrum.uni-wuerzburg.de. The work-bench offers nucleotide pattern analysis for promotor and regulatory element detection (tardigrade specific; nrdb) as well as rapid COG search for function assignments including species-specific repositories of all analysed data.

**Conclusion:**

Different protein clusters and regulatory elements implicated in tardigrade stress adaptations are analysed including unpublished tardigrade sequences.

## Background

Tardigrades are small metazoans resembling microscopic bears ("water-bears", 0.05 mm to 1.5 mm in size) and live in marine, freshwater and terrestrial environments, especially in lichens and mosses [1-3]. They are a phylum of multi-cellular animals capable of reversible suspension of their metabolism and entering a state of cryptobiosis [4,5]. A dehydrated tardigrade, known as anhydrobiotic tun-stage [6,7], can survive for years without water. Moreover, the tun is resistant to extreme pressures and temperatures (low/high), as well as radiation and vaccuum [8-13].

Well known species include *Hypsibius dujardini *which is an obligatory parthenogenetic species [14]. The tardigrade *H. dujardini *can be cultured continuously for decades and can be cryopreserved. It has a compact genome, a little smaller than that of *Caenorhabditis elegans *or *Drosophila melanogaster*, and the rate of protein evolution in *H. dujardini *is similar to that of other metazoan taxa [15]. *H. dujardini *has a short generation time, 13-14 days at room temperature. Embryos of *H. dujardini *have a stereotyped cleavage pattern with asymmetric cell divisions, nuclear migrations, and cell migrations occurring in reproducible patterns [15]. Molecular data are sparse but include the purinergic receptor occuring in *H. dujardini *[16].

*Milnesium tardigradum *is an abundant and ubiquitous terrestrial tardigrade species in Europe and possibly worldwide [17]. It has unique anatomy and motion characteristics compared to other water bears. Most water bears prefer vegetarian food, *M. tardigradum *is more carnivorous, feeding on rotifers and nematodes. The animals are really tough and long-living, one of the reasons why *M. tardigradum *is one of the best-studied species so far.

Questions of general interest are: How related are tardigrade proteins to each other? Which protein families provide tardigrade-specific adaptations? Which regulatory elements influence the mRNA stability? Starting from all published tardigrade sequences as well as 607 unpublished new sequences from *Milnesium tardigradum*, we analyse tardigrade specific clusters of related proteins, functional protein clusters and conserved regulatory elements in mRNA mainly involved in mRNA stability. The different clusters and identified motifs are analysed and discussed, all data are also available as a first anchor to study specific adaptations of tardigrades in more detail (Tardigrade workbench). Furthermore, the tardigrade analyzer, a sequence server to analyse individual tardigrade specific sequences, is made available. It will be regularly updated to include new tardigrade sequences. It has a number of new features for tardigrade analysis not available from standard servers such as the NIH Entrez system [18]: several new species-specific searches (*Echiniscus testudo*, *Tulinus stephaniae*), additional new sequence information (*M. tardigradum*) and pattern-searches for nucleotide sequences (including pattern search on non-redundant protein database, NRDB). An easy search for clusters of orthologous groups (COG, [19]) different from the COGnitor tool [20] allowing tardigrade specific COG and eukaryotic COG (KOG) searches is also available.

Furthermore, a batch mode allows a rapid analysis of up to 100 sequences simultaneously when uploaded in a file in FASTA format (for tardigrade species or NRDB).

Two fifths of the tardigrade sequences cluster in longer protein families, and we hypothesise for a number of these that they are implicated in the unique stress adaptation potential of tardigrades. We find also ten tardigrade specific clusters. The unique tardigrade adaptions are furthermore indicated by a number of functional COGs and KOGs identified here, showing a particular emphasis on the protection of proteins and DNA. RNA read out is specifically regulated by several motifs for mRNA stability clearly overrepresented in tardigrades.

## Results and Discussion

We analysed all publicly available tardigrade sequences (status 9^th ^of April 2009) as well as 607 unpublished *M. tardigradum *sequences from our ongoing transcriptome analysis.

### Major tardigrade protein clusters of related sequence-similar proteins

All available tardigrade sequences were clustered by the CLANS algorithm [21]. Interestingly, 39.3 % of the predicted proteins (mainly EST-based predictions) cluster in just 58 major families, each with at least 20 sequences [see additional file 1: Table S1]. These include 4,242 EST sequences from a total of 10,787.

Using these clusters, a number of tardigrade-specific adaptations become apparent (Table 1 [and additional file 1: Table S1]): the clusters include elongation factors (cluster 12), ribosomal RNAs and proteins (cluster 1, 4, 32 and 56) which are part of the transcriptional or translational machinery. Cluster 5 (chitinase binding domain [22]) could provide membrane and structural reorganization or immune protection (e.g. fungi) according to homologous protein sequences characterized in other organisms. Other clusters show protein families related to the tardigrade stress adaptation potential, e.g. ubiquitin-related proteins (cluster 14; maybe stress-induced protein degradation) and cytochrome oxidase-related proteins (cluster 2, suggested to be involved in respiratory chain).

**Table 1:** CLANS clusters of sequence similar proteins in published tardigrade sequences^1^

Number/color	Cluster description	Sequences/percentage
2	Cytochrome c oxidase like (subunit 1, EC 1.9.3.1)	425 (3.94%)
3	Uncharacterized protein U88/Glycosyltransferase 8 family	302 (2.80%)
5	Proteins containing a Chitin binding domain	191 (1.77%)
6	Proteins containing an IBR/Neuroparsin/DUF1096 domain	189 (1.75%)
7	Fatty-acid binding protein (FABP) family	127 (1.18%)
8	TSP^2 ^(remote homology to Sericin 1)	126 (1.17%)
9	Proteins containing a RNA polymerase Rpb3/Rpb11 dimerisation domain	92 (0.85%)
10	Metallothionein superfamily (Type 15 family./Thioredoxin like)	84 (0.78%)
12	GTP-binding elongation factor family. EF-Tu/EF-1A sub- family	79 (0.72%)
13	GST superfamily. Sigma family	78 (0.70%)
14	Ubiquitin family	75 (0.69%)
15	Cathepsin family (EC 3.4.22.-)	74 (0.67%)
16	Carboxypeptidase A inhibitor like	72 (0.64%)
17	Trichohyalin/Translation initiation factor like	69 (0.60%)
18	TSP^2^	65 (0.57%)
19	TSP^2^	61 (0.56%)
20	RNA/DNA-binding proteins	60 (0.55%)
	...	
23	Small Heat Shock Protein (HSP20) family	53 (0.47%)
24	Diapause-specific proteins	51 (0.44%)
	...	
38	LEA type 1 family proteins	31 (0.28%)
	...	

^1 ^Shown are the number of proteins found for the specified cluster, their percentages and the corresponding cluster number in Figure 1. The full Table with all clusters and their color code matching to Figure 1 is given in [additional file 1: Table S1]. Clusters 1, 4 and 11 contain rRNA and are given in [additional file 1].

^2 ^Tardigrade specific protein cluster

Moreover, proteins responsible for protein degradation (cluster 15) were found as well as proteins regulating peptidases (cluster 16). Cluster 23 consists of 53 heat shock proteins which are involved in many stress response reactions [23]. Few diapause specific proteins (cluster 24) are known from other animals. Diapause is a reversible state of developmental suspension. It is observed in diverse taxa, from plants to animals, including marsupials and some other mammals [24] as well as insects (associated molecular function varies but involves calcium channel inhibition [25]) and should here support the tun formation or regulate other (e.g. developmental) metabolic inactive states. Furthermore, proteins involved in storage or transportation of fatty acids also seem to be important (cluster 31, [26]). Late embryogenesis abundant (LEA) protein expression seems to be linked to desiccation stress and the acquisition of desiccation tolerance in organisms [27] e.g. nematodes [28,29] and rotifers [30]. Thirty-one LEA type 1 family proteins were found in cluster 38.

LEA proteins are wide-spread among plants and synthesized in response to certain stresses [31,32]. The LEA type 1 family is well known in higher plants (rice, maize, carrots) to be synthesized during late embryogenesis and in ABA stress response. It includes desiccation-related protein PCC3-06 of *Cratersostigma plantagineum*. LEA type 1 family occurs in bacteria (e.g. *Haemophilus influenzae, Deinococcus radiodurans*), but is atypical for animals. However, this is an animal example where LEA family type 1 is well represented and forms a full cluster.

Moreover, ten clusters (8, 18, 19, 30, 33, 35, 37, 42, 51, 55) consist of proteins which seem to be specific for tardigrades. These show no significant homology to known proteins.

### Functional clusters of stress-specific adaptations present in tardigrades

To gain a systematic overview of involved tardigrade functions, all available tardigrade sequences were classified species-specific according to COG functional category [19,20] as well as according to COG number and molecular function encoded. Note that in this section "protein" implies one type of protein. A COG or KOG comprises often several sequences from different tardigrades. Prokaryotic (COG) and eukaryotic (KOG) gene clusters were compared (Table 2; details on the WEB http://waterbear.bioapps.biozentrum.uni-wuerzburg.de/). Again, several tardigrade-specific adaptations stand out, e.g. highly represented COGs regulate translation elongation factor and sulfate adenylate transferase and a strong ubiquitin system. There are many cysteine proteases (21 proteins). For redox protection there are 14 thioredoxin-domain containing proteins and 75 Heme/copper-type cytochrome/quinol-like proteins as well as ubiquinone oxidoreductase subunits (15 proteins). There are ten proteins involved in seleno-cysteine specific translation [33,34]. In eukaryotes, selenoproteins show a mosaic occurrence, with some organisms, such as vertebrates and algae, but notably also tardigrades, having dozens of these proteins, while other organisms, such as higher plants and fungi, having lost all selenoproteins during evolution [34]. Membrane GTPases (25 proteins) are often of Lep A (leader peptidase [35]) type in tardigrades. In general, members of the GTPase superfamily regulate membrane signaling pathways in all cells. However, LepA, as well as NodO, are prokaryotic-type GTPases very similar to protein synthesis elongation factors but apparently have membrane-related functions [35]. It is interesting to observe this prokaryotic-type GTPase in tardigrades. We suggest that it will have similar function as known in other organisms and thus ensure protein translation (elongation factor) coupled to membrane integrity and possibly cytoskeletal rearrangement which would again boost the tardigrade resistance to stress.

**Table 2:** Highly represented protein functions in Tardigrades (COGs and KOGs).

*Information from COG clusters*^1^:
Information storage and processing
75 Translation elongation factor EF-1 (COG5256)
64 GTPases - translation elongation (COG0050)
58 Peptide chain release factor RF-3 (COG4108)
Cellular processes and signaling
31 Ubiquitin (COG5272)
25 Membrane GTPase LepA (COG0481)
21 Cysteine protease (COG4870)
Metabolism
75 Heme/copper-type cytochrome/quinol oxidases (COG0843)
67 GTPases - Sulfate adenylate transferase (COG2895)
Poorly characterized
11 Dehydrogenases with different specificities (COG1028)
11 Uncharacterized homolog of Blt101 (COG0401)
*Information from KOG clusters*^1^:
Information storage and processing
77 Translation elongation factor EF-1 (KOG0052)
71 Polypeptide release factor 3 (KOG0459)
70 Elongation factor 1 alpha (KOG0458)
53 Mitochondrial translation elongation factor Tu (KOG0460)
Cellular processes and signaling
52 Glutathione S-transferase (KOG1695)
46 Cysteine proteinase Cathepsin L (KOG1543)
34 Apolipoprotein D/Lipocalin (KOG4824)
31 Cysteine proteinase Cathepsin F (KOG1542)
Metabolism
78 Cytochrome c oxidase (KOG4769)
74 Fatty acid-binding protein FABP (KOG4015)
Poorly characterized
31 Ubiquitin and ubiquitin-like proteins (KOG0001)
16 GTPase Rab18, small G protein superfamily (KOG0080)
15 Ras-related GTPase (KOG0394)
15 GTPase Rab21, small G protein superfamily (KOG0088)

^1 ^Detailed data and all COG/KOG numbers are given on the WEB page http://waterbear.bioapps.biozentrum.uni-wuerzburg.de/cgi-bin/cog_stat.pl. Summarized here are the functions of those clusters of orthologous groups (COGs) occurring particularly often or suggesting tardigrade specific adaptations.

The KOGs show similar highly represented families and adaptations. Abberant proteins are rapidly recognized by ubiquitination-like proteins (220 proteins) and ubiquitin-ligase related enzymes (71 proteins) as well as proteasome regulatory subunits (85 proteins). For protein protection and refolding disulfide isomerases (26 proteins) and cyclophilin type peptidyl-prolyl cis-trans isomerases (43 proteins; KOG 0879-0885) are available. Connected to redox protection are also thirty AAA+type ATPases and three peroxisome assembly factor 2 containing proteins (KOG0736). This broad effort in protein protection is further supported by molecular chaperones (HSP70, mortalins and other; total of 50 proteins) and chaperonin complex components (32 proteins; KOG0356-0364). There are six superoxide dismutases and six copper chaperons for thioredoxins (37 proteins), glutaredoxin-like proteins (nine) and ten thiodisulfide isomerases as well as 52 glutathione-S-transferases. We found 22 hits to helicases. Tardigrade DNA protection is represented by 52 proteins of the molecular chaperone DNA J family: proteins of the DNA J family are classified into 3 types according to their structural domain decomposition. Type I J proteins compose of the J domain, a gly-rich region connecting the J domain and a zinc finger domain, and possibly a C-terminal domain. Type II lacks the Zn-finger domain and type III only contains the J domain [36,37]. The latter two are referred to as DnaJ-like proteins. Analysis of the domains present in tardigrade proteins by SMART [38] and Pfam [39] searches reveals only the J domain and in some cases a transmembrane region, identifying them as type III DnaJ-like proteins. For further information on these COGs/KOGs see Table 3.

**Table 3:** Identified DnaJ-family COGs/KOGs in Tardigrades and *Milnesium tardigradum*^1^.

			present in	
KOG/COG number	COG distribution	COG name	Tardigrades	M. tardigradum*
COG0484		DnaJ-class molecular chaperone with C-terminal Zn finger domain	5	
COG2214		DnaJ-class molecular chaperone	8	
KOG0550	A-DH-P-	Molecular chaperone (DnaJ superfamily)	3	2
KOG0691	ACDHYP-	Molecular chaperone (DnaJ su perfamily)	7	2
KOG0712	ACDHYPE	Molecular chaperone (DnaJ su perfamily)	8	2
KOG0713	ACDH---	Molecular chaperone (DnaJ su perfamily)	5	1

^1^Shown are the number of proteins found for the specified COG/KOG number, the KOG distribution of the KOG in different eukaryotic species (see abbreviations) and the COG/KOG annotation.

Abbreviations: A *Arabidopsis thaliana*, C *Caenorhabditis elegans*, D *Drosophila melanogaster*, H *Homo sapiens*, Y *Saccharomyces cerevisiae*, P *Schizosaccharomyces pombe*, E *Encephalitozoon cuniculi*. *These include specific unpublished data from ongoing work on *M. tardigradum*

Moreover, undesired proteins can be rapidly degraded by cathepsin F-like proteins (31 proteins) or L-like proteins (46 proteins). There are several calcium-dependent protein kinases (25 proteins; KOG0032-0034) and actin-bundling proteins. According to this observation calcium signaling should be implicated in adaptive rearrangement of the cytoskeleton during tardigrade rehydration. The cytoskeleton is a key element in the organisation of eukaryotic cells. It has been described in the literature that the properties of actin are modulated by small heat-shock proteins including a direct actin-small heat-shock protein interaction to inhibit actin polymerization to protect the cytoskeleton [40,41] (compare with the CLANS cluster 24 (Diapause proteins) found in the above analysis).

Translation in tardigrades includes polypeptide release factors (71 proteins) and proteins for translation elongation (77 proteins). There are about 80 GTP-binding ADP-ribosylation factors. The secretion system and Rab/Ras GTPases are fully represented (183 proteins). Seventeen tubulin anchor proteins show that the cytoskeleton is well maintained. Finally, we find 14 TNF-associated factors and 34 apolipoprotein D/lipocalin proteins.

### Typical motifs in tardigrade mRNAs

The regulatory motif search showed a number of known regulatory RNA elements involved in tardigrade mRNA regulation (Table 4 for *H. dujardini *and *M. tardigradum*). Certainly it can not be formally ruled out that some of these elements work in a tardigrade modified way. Similarly, there are probably further patterns which are tardigrade specific, but not detected with the UTRscan software [42] applied for analysis.

**Table 4:** Regulatory elements in *Hypsibius dujardini*^1 ^and *Milnesium tardigradum*^2 ^mRNA sequences.

Motiv	*Hypsibius dujardini*	*Milnesium tardigradum*
15-LOX-DICE	1528 (1269)^3^	46 (45)^3^
ADH DRE	60 (58)^3^	1 (1)^3^
BRE	1(1)^3^	--
Brd-Box	152 (149)^3^	28 (22)^3^
CPE	21 (21)^3^	15 (15)^3^
Elastin G3A	1 (1)^3^	--
GLUT1	1 (1)^3^	--
GY-Box	406 (372)^3^	21 (21)^3^
IRE	1 (1)^3^	--
IRES	1353 (1353)^3^	90 (90)^3^
K-Box	469 (447)^3^	35 (33)^3^
SECIS-1	1 (1)^3^	--
SECIS-2	6 (6)^3^	--
TGE	5 (5)^3^	1 (1)^3^
TOP	50 (50)^3^	1 (1)^3^

^1 ^We considered 5,378 ESTs in *H. dujardini*. ^2 ^We considered 607 ESTs in *M. tardigradum*. ^3 ^The number of hits is followed by the number of mRNAs with this hit in brackets to indicate multiple hits.

The RNA elements found include the lox-P DICE element [43] in *H. dujardini *as top hit with as many as 1,269 ESTs (23.6% of all *H. dujardini *EST sequences). The cytidine-rich 15-lipoxygenase differentiation control element (15-LOX DICE, [44]) binds KH domain proteins of the type hnRNP E and K (stronger in multiple copies), mediating mRNA stabilization and translational control [43].

Furthermore, a high number of mRNAs contains K-Boxes (cUGUGAUa, [45]) and brd Boxes (AGCUUUA, [46]). All these elements are involved in mRNA storage and mRNA stability. These two elements are potential targets for miRNAs as shown in *Drosophila melanogaster *[47].

However, in the two tardigrade species compared, only 16 of 30 well known RNA elements are found, suggesting a clear bias in tardigrade mRNA regulation. For example, the widely used AU rich elements in higher organisms [42] such as vertebrates are absent in tardigrades [see additional file 1].

Regulatory elements in tardigrade mRNA are probably important for their adaptation, in particular to support transformation to tun stage and back to active stage again. The list of RNA elements found can be compared for instance to our data on regulatory elements in human anucleate platelets [48] where mRNAs have to be stockpiled for the whole life of the platelet. Due to this comparatively long life, a long mRNA untranslated region is important in these cells. The same should apply to tardigrade mRNAs, since their average UTR is predicted to be long. A different stock-piling scenario occurs in unfertilized eggs, but due to developmental constraints, here localization signals are often in addition important for developmental gradients. We tested for these in tardigrades but did not find a high representation of localization motifs.

### Web-tool tardigrade analyzer

We created a convenient platform to allow rapid sequence comparisons of new protein sequences, in particular from new sequencing efforts in tardigrades, to our database by applying rapid heuristic local alignment using BLAST [49] and allowing to search in selected species.

A batch mode allows the analysis of up to 100 sequences simultaneously when uploaded in a file in FASTA format. Output data are displayed according to an enhanced BLAST output format with graphical illustrations. Low expected E-values result for searches using the option of our tardigrade specific databases: a more specific smaller database reduces the probability of false positives. As an alternative for general sequence analysis, a search against the non-redundant database of GenBank can be performed. This takes more computational power and yields higher E-values, however, it identifies functions for most sequences. An additional useful feature is to scan all available data for peptide motifs or PROSITE signatures using a "pattern" module [additional file 1: Fig. S1] or assign potential functions by COGs [19]. The first is helpful to recognize tardigrade proteins in cases where the tardigrade sequence has diverged far, and only critical residues for function are still conserved as motif signatures. It can also be applied to search for regulatory RNA motifs such as polyadenylation sites (e.g. AAUAAA or AAUUAA) or recognize promotor modules such as the glucocorticoid receptor element (GRE; palindromic pattern: AGAACAnnnTGTTCT). For this purpose, both, the tardigrade sequences and the non redundant database can be searched (e.g. to look for stress-specific regulatory RNA elements; [additional file 1, Fig. S2]).

**Figure 1:**
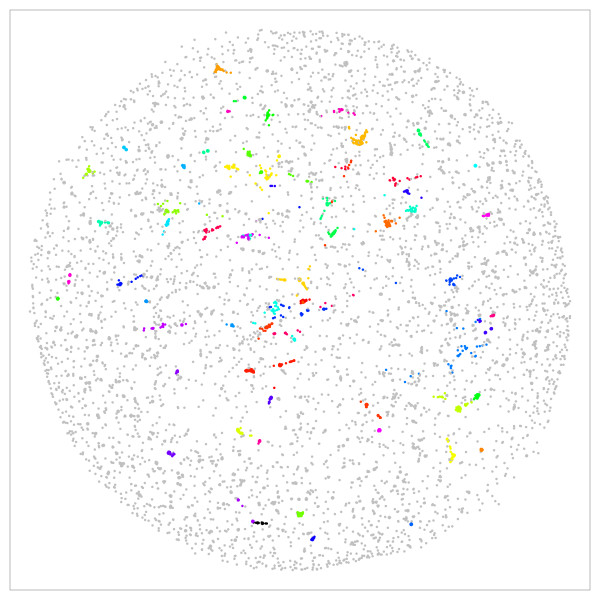
**Functional clusters by CLANS of sequence related proteins in tardigrades**. All available [see additional file 1: Figure S5] tardigrade protein sequences were clustered in a 3D sphere according to their sequence distance and were projected to the paper plane. Individual protein functions are colored [for color code see additional file 1: Table S1] and all listed in Table 1. Functional clusters appear as patches of an individual color. Color code and clusters can be interactively examined at the Tardigrade workbench http://waterbear.bioapps.biozentrum.uni-wuerzburg.de and are given in [additional file 1 Table S1]. figure1.pdf

Interestingly, this nucleotide (RNA or DNA) specific option is not available on some common servers, e.g. the PHI-BLAST [50] server at NIH. Further options include a user-defined database [additional file 1: Fig. S3] and interactively animated stress clusters (Figure 1).

The tool http://waterbear.bioapps.biozentrum.uni-wuerzburg.de/ allows rapid searches for tardigrade specific sequences, e.g. molecular adaptations against stress [see additional file 1 for screenshots and a tutorial]. For instance, a search for trehalase sequences shows no trehalase mRNA in the *H. dujardini *sequences. In contrast, there are several heat shock proteins in tardigrades, an example is HSP90 proteins (identified by sequence similarity as well as by a pattern hit based approach using the PROSITE entry PS00298 with the signature Y-x- [NQHD]- [KHR]- [DE]- [IVA]-F- [LM]-R- [ED]; Table 5). Specific COGs are also rapidly assigned for any desired sequence. This includes the option to map the query sequence of interest to any of the known tardigrade specific COGs. Furthermore, nucleotide patterns such as mRNA polyadenylation sites are rapidly identified e.g. in *H. dujardini *mRNAs [additional file 1: Fig. S4]. Similarly, other mRNA 3'UTR elements can be identified, e.g. AU rich sequences mediating mRNA instability or regulatory K-boxes (motif cUGUGAUa, [45]) in tardigrades.

**Table 5:** HSP90 proteins identified in *Hypsibius dujardini *using the Tardigrade analyzer^1^.

Hit	Predicted function/name (Tardigrade analyzer)	Pattern matched	Start	End
			position	
gi:37213462	hsp90^2^	YSNKEIFLRE	68	77
gi:37213713	hsp90^2^	YSNKEIFLRE	70	79

^1^These are hits using the pattern hit option and the heat shock protein PROSITE entry PS00298 for pattern generation and recognition. The pattern has the signature of Y-x- [NQHD]- [KHR]- [DE]- [IVA]-F- [LM]-R- [ED]. ^2 ^Predicted similarity to Q7PT10 (HSP83 ANOGA) from Swissprot

### Implications

Tardigrades show a surprising large amount of related sequences. Certainly, one has to correct for a few genes sequenced from many lineages for phylogenetic studies in tardigrades (cytochrome c, rRNA etc.) However, despite this, a number of tardigrade-specific clusters still remain. Furthermore, Table 1 shows that most of the annotated clusters are stress-related.

Looking at specific protein functions, both COG and KOG proteins show that tardigrades spend an extraordinary effort in protein protection, turnover and recycling as well as redox protection. Some other specific adaptations become apparent also from Table 2, but the complete extent of these adaptations is unclear given the limited sampling of available tardigrade sequences. Furthermore, protection of DNA is critical as it has been shown that tardigrade tuns accumulate DNA damage which first has to be repaired before resurrection occurs [51,52]. Taking this into consideration, DNA J proteins were investigated in more detail since proteins of this family are well represented in tardigrades, including several COGs and KOGs. Several data underline the extremely high resistance of tardigrades to temperature, pressure and radiation as well as a high repair potential regarding DNA [11,51]. Thus, we suggest that the high repair potential is also mediated by this well represented protein family. Phylogenetic analysis (Table 3) shows that these proteins are represented by several KOGs as well as the classic COGs in tardigrades. In particular, the first three KOG families are also used in *M. tardigradum*, where extreme stress tolerance requires strong repair mechanisms [17]. Furthermore, all these tardigrade proteins in Table 3 are small, having neither zinc-finger domains nor low complexity regions, but instead consisting of single DNA J domains which would always place them in type I (subfamily A) of DNA-J like proteins. This suggests that the direct interaction with DNA-J like proteins is the key molecular function.

Finally, we could show that there are 16 regulatory elements used in tardigrade mRNA, while a number of other elements known from higher eukaryotic organisms and vertebrates is not used. It is interesting to note that the elements often used in tardigrades are all involved in regulation of mRNA stability. Thus, they may be implicated in stage switching, as presumably in the initial phases of the tun awakening or tun formation, new supply of mRNA is turned off and instead regulation of synthesized mRNA becomes important.

In addition, and for further research we supply the web tool tardigrade analyzer. There are a number of alternative tools available, e.g. from NCBI http://www.ncbi.nlm.nih.gov/. However, we offer some species-specific searches not available from these sources as well as RNA and promotor pattern search (not only for tardigrades but also for NRDB; not available from NIH). Furthermore, there are functional COG prediction as well as new, unpublished tardigrade sequences from *M. tardigradum*, all above reported data including the reported sequences and detailed functional clusterings as well as regular server updates. A better understanding of the survival mechanisms in these organisms will lead to the development of new methods in several areas of biotechnology. For example, preservation of biological materials *in situ*, macromolecules and cells from non-adapted organisms [53]. This is, of course, only a first and very general overview on potential tardigrade specific adaptations, more species-specific data will be considered as more information becomes available.

## Conclusion

Tardigrade genomes invest in stress-specific adaptations, this includes major sequence related protein clusters, functional clusters for stress as well as specific regulatory elements in mRNA. For further tardigrade genome analysis we offer the tardigrade workbench as a flexible tool for rapid and efficient analysis of sequence similarity, protein function and clusters, COG membership and regulatory elements.

## Methods

### Tardigrade-sequences

The cosmopolitan eutardigrade species *M. tardigradum *Doyére 1849 (Apochela, Milnesidae) was cultured. Tardigrades were kept and reared on petri dishes (diameter: 9.4 cm) filled with a small layer of agarose (3 %) (peqGOLD Universal Agarose, peqLAB, Erlangen, Germany) and covered with spring water (Volvic™ water, Danone Waters Deutschland, Wiesbaden, Germany) at 20 ± 2°C and a light/dark cycle of 12 h. Rotifers *Philodina citrina *and nematodes *Panagrellus *sp. were provided as food source, juvenile tardigrades were also fed with green algae *Chlorogonium elongatum*. For all experiments adult animals in good physical condition were taken directly from the culture and starved for three days to avoid preparation of additional RNA originating from not completely digested food in the intestinal system. For an overview of RNAs present both in active and tun stage we used a mixture of the same number of animals.

Total RNA extraction was performed using the QIAGEN RNeasy^®^Mini kit (Qiagen, Hilden, Germany). The cDNA synthesis was reversed transcribed using 1 *μ*g total RNA by the Creator™ SMART™ cDNA Library Construction Kit (Clontech-Takara Bio Europe, France). The resulting cDNA was amplified following the manufacturers protocol and cloned into pDNR-Lib cloning vector. The resulting plasmids were used to transform *Escherichia coli *by electroporation. Sequencing of the cDNA-library was done by ABI 3730XL capillary sequencer (GATC Biotech AG, Konstanz, Germany). All obtained EST sequences were deposited with Genbank including dbEST databank.

Nucleotide sequences from other tardigrades were collected from Genbank. For *H. dujardini*, the best represented species, we composed 5,235 ESTs. We stored *H. dujardini *as well as all published sequences of other tardigrade species (e.g. *T. stephaniae*, *E. testudo*, *M. tardigradum*, *R. coronifer*) in a database (10,787 sequences including translated sequences, details in [additional file 1], status on April, 2009).

### CLANS clustering

For a systematic overview on tardigrade specific adaptations we first clustered all published tardigrade nucleotide sequences into functional clusters (Figure 1) using the Cluster analysis of sequences (CLANS) algorithm [21]. All sequences were clustered in 3D space using 0.001 as an E-value cut-off for TBLASTX all-against-all searches. [additional file 1: Fig. S4].

### Identification of regulatory elements

For this the ESTs of *H. dujardini *and *M. tardigradum *were systematically screened using the software UTRscan [42]. This software screens 30 regulatory elements for RNA regulation with a focus on 3' UTR elements and stability of mRNA. The default settings for batch mode were used and all reported elements were collected.

### COG clustering and identification

In order to acquire a systematic overview of the functionalities, we used the latest version of COG/KOG databases ftp://ftp.ncbi.nih.gov/pub/COG and the BLAST hits from both nucleotide search and protein search were clustered according to their COG ID. Searches were carried out in parallel on all the tardigrade species including *M. tardigradum*, *H. dujardini*, *E. testudo*, *T. stephaniae *and *R. coronifer*. The results are summarized in a table shown in the tardigrade analyzer, the background color from cold to warm (blue to red) indicates the cluster size, which enables an easy comparison. Moreover, users are allowed to click the COG ID and the hit number. The server then reports the corresponding sequence ID, description, conservation and the homologous entries recorded in the database. The server with its data is automatically updated bi-monthly according to the latest tardigrade databases.

### Tardigrade workbench

The tardigrade workbench is implemented in Perl using the Bioperl modules [54]. NCBI BLAST program of 2.2.17 is involved in the software package. A database of Postgresql 8.1.9 is applied to manage the tardigrade entries so as to accelerate the searching queried by investigators. The COG cluster information is automatically updated each week and warehoused on the server. In addition, the run of tardigrade workbench requires an Apache server, a linux system of at least 2 GB memory is highly recommended.

## Authors' contributions

FF did tardigrade protein data analysis including CLANS clustering and RNA motif analysis. CL established the current version of the tardigrade workbench including programming new routines, data management and nucleotide motif analysis. AS did the initial setup of the server, of the virtual ribosome and the CLANS clustering. DB, JE, MS and MF participated in tardigrade data analysis. TM gave expert advice and input on statistics, RS gave expert advice on tardigrade physiology and zoology. TD led and guided the study including analysis of data and program, supervision, and manuscript writing. All authors participated in the writing of the manuscript and approved the final version.

## Supplementary Material

Additional file 1 **Additional Tables and Figures**. The file contains seven additional figures and two additional tables. One of these tables summarizes annotation and different identifiers for 607 new EST sequences from *Milne-sium tardigradum*. Click here for file
