# Supplementary material for: Tardigrade workbench: comparing stress-related proteins, sequence-similar and functional protein clusters as well as RNA elements in tardigrades
Source: BMC Genomics. 2009 Oct 12;10:469. doi: 10.1186/1471-2164-10-469 (PMC2768748; doi:10.1186/1471-2164-10-469)
Supplement: Additional file 1 — Additional Tables and Figures. The file contains seven additional figures and two additional tables. One of these tables summarizes annotation and different identifiers for 607 new EST sequences from Milne-sium tardigradum. [file 1471-2164-10-469-S1.PDF]

Tardigrade workbench: Comparing stress-related proteins, sequence-similar and functional protein clusters as well as RNA elements in tardigrades supplemental material

Frank Förster[1,§], Chuanguang Liang[1,§], Alexander Shkumatov[2,§], Daniela Beisser[1], Julia C. Engelmann[1], Martina Schnölzer[3], Marcus Frohme[4], Tobias Müller[1], Ralph O. Schill[5], Thomas Dandekar*[1]

[1]Dept. of Bioinformatics, Biocenter University of Würzburg, 97074 Würzburg, Germany
[2]EMBL, Hamburg Outstation, Notkestrasse 85, 22603 Hamburg, Germany
[3]Functional Proteome Analysis, German Cancer Research Center, Im Neuenheimer Feld 580, 69120 Heidelberg, Germany
[4]University of Applied Sciences, Bahnhofstraße 1, 15745 Wildau, Germany
[5]Dept. of Zoology, Institute for Biology, Universität Stuttgart, 70569 Stuttgart, Germany
[§] these authors contributed equally

Email: Frank Förster - frank.foerster@biozentrum.uni-wuerzburg.de; Chuanguang Liang - liang@biozentrum.uni-wuerzburg.de; Alexander Shkumatov - ashkumat@embl-hamburg.de; Daniela Beisser - daniela.beisser@biozentrum.uni-wuerzburg.de; Julia C. Engelmann - julia.engelmann@klinik.uni-regensburg.de; Martina Schnölzer - m.schnoelzer@dkfz-heidelberg.de; Marcus Frohme - mfrohme@thf-wildau.de; Tobias Müller - tobias.mueller@biozentrum.uni-wuerzburg.de; Ralph O. Schill - ralph.schill@bio.uni-stuttgart.de; Thomas Dandekar - dandekar@biozentrum.uni-wuerzburg.de;

*Corresponding author

Supplementary Material

Protocol of EST translation to derive the protein reading frames for the tardigrade specific protein database

Currently, there are 10,701 nucleotide sequences (5,235 ESTs, 1,043 core nucleotides, 1,063 GSS nucleotides, 3,360 ESTs from trace archive) and 228 protein sequences available in NCBI databases (stand January 2009). In order to understand molecular mechanisms underlying cryptobiosis, we performed an automated translation and ORF detection.

The procedure is depicted in Figure S6. Nucleotide sequences coding for known proteins were identified using BLASTX [1] against the UniProtKB/SwissProt-, UniProtKB/TrEMBL- and NR-database [2, 3] and in parallel a six frame translation using virtual ribosome [4] followed by a HMMER search [5] against the PFAM-database [6]. For these sequences the ORFs resulting in hits against BLAST or PFAM were extracted. Sequences without result in BLASTX were searched against the next more extensive database. Finally sequences without significant hits were translated into six frames and all ORFs containing 100 or

more amino acids were extracted. If no ORF had a length of 100 or more amino acids, we took the longest ORF.

To exclude sequences containing rRNA, we identified them using a BLASTN against a database of eukaryotic rRNAs [7]. These sequences were substracted from translated sequences.

The last step in translation process was to collapse sequences resulting in the same ORF for BLASTX and PFAM search. Finally we obtained 8,334 protein sequences.

Brief tutorial to the Tardigrade analyzer (http://waterbear.bioapps.biozentrum.uni-wuerzburg.de)

Users access the searching functions in the top navigation-bar of the pages. BLAST search can be conducted both upon protein and nucleotide sequences specific for tardigrades, or on the non-redundant database from Genbank (left), queries support all standard sequence formats and parameters (E-value, filter and matrix). The output report is generated in HTML. There are two types of grammars available for pattern searches:

- Perl-format regular expressions to state flexible motif variation on protein and nucleotide sequences. This includes searches for regulatory elements.
- Prosite motif expressions according to the Expasy database (www.expasy.ch). Regarding the Prosite database support, users may apply the Prosite signatures directly in the server.

Examples and further tutorials are online available within the querying page (via “HELP DOC” button and example link).

References

1. Altschul SF, Madden TL, Schäffer AA, Zhang J, Zhang Z, Miller W, Lipman DJ: **Gapped BLAST and PSI-BLAST: a new generation of protein database search programs.** *Nucleic Acids Res* 1997, **25**:3389–3402.
2. UniProt Consortium: **The universal protein resource (UniProt).** *Nucleic Acids Res* 2008, **36**:D190–D195.
3. Pruitt KD, Tatusova T, Maglott DR: **NCBI reference sequences (RefSeq): a curated non-redundant sequence database of genomes, transcripts and proteins.** *Nucleic Acids Res* 2007, **35**:D61–D65.
4. Wernersson R: **Virtual Ribosome–a comprehensive DNA translation tool with support for integration of sequence feature annotation.** *Nucleic Acids Res* 2006, **34**:W385–W388.
5. Durbin R: *Biological sequence analysis: Probabilistic models of proteins and nucleic acids.* Cambridge university press 1998.
6. Finn RD, Mistry J, Schuster-Böckler B, Griffiths-Jones S, Hollich V, Lassmann T, Moxon S, Marshall M, Khanna A, Durbin R, Eddy SR, Sonnhammer ELL, Bateman A: **Pfam: clans, web tools and services.** *Nucleic Acids Res* 2006, **34**:D247–D251.

7. Pruesse E, Quast C, Knittel K, Fuchs BM, Ludwig W, Peplies J, Glöckner FO: **SILVA: a comprehensive online resource for quality checked and aligned ribosomal RNA sequence data compatible with ARB.** *Nucleic Acids Res* 2007, **35**:7188–7196.

8. Frickey T, Lupas A: **CLANS: a Java application for visualizing protein families based on pairwise similarity.** *Bioinformatics* 2004, **20**:3702–3704.

9. Gaudermann P, Vogl I, Zientz E, Silva FJ, Moya A, Gross R, Dandekar T: **Analysis of and function predictions for previously conserved hypothetical or putative proteins in Blochmannia floridanus.** *BMC Microbiol* 2006, **6**:1.

Figures

Figure S1: Tardigrade analyzer input mask and options.

Shown are direct WEB Server input masks at the Tardigrade analyzer (http://waterbear.bioapps.biozentrum.uni-wuerzburg.de/). BLAST search (left page) and pattern-matching search (right page) as well as the COG search module (not shown; choose from the menu bar on top) include different options for users to specify the query sequence formats, databases and species, programs and program parameters.

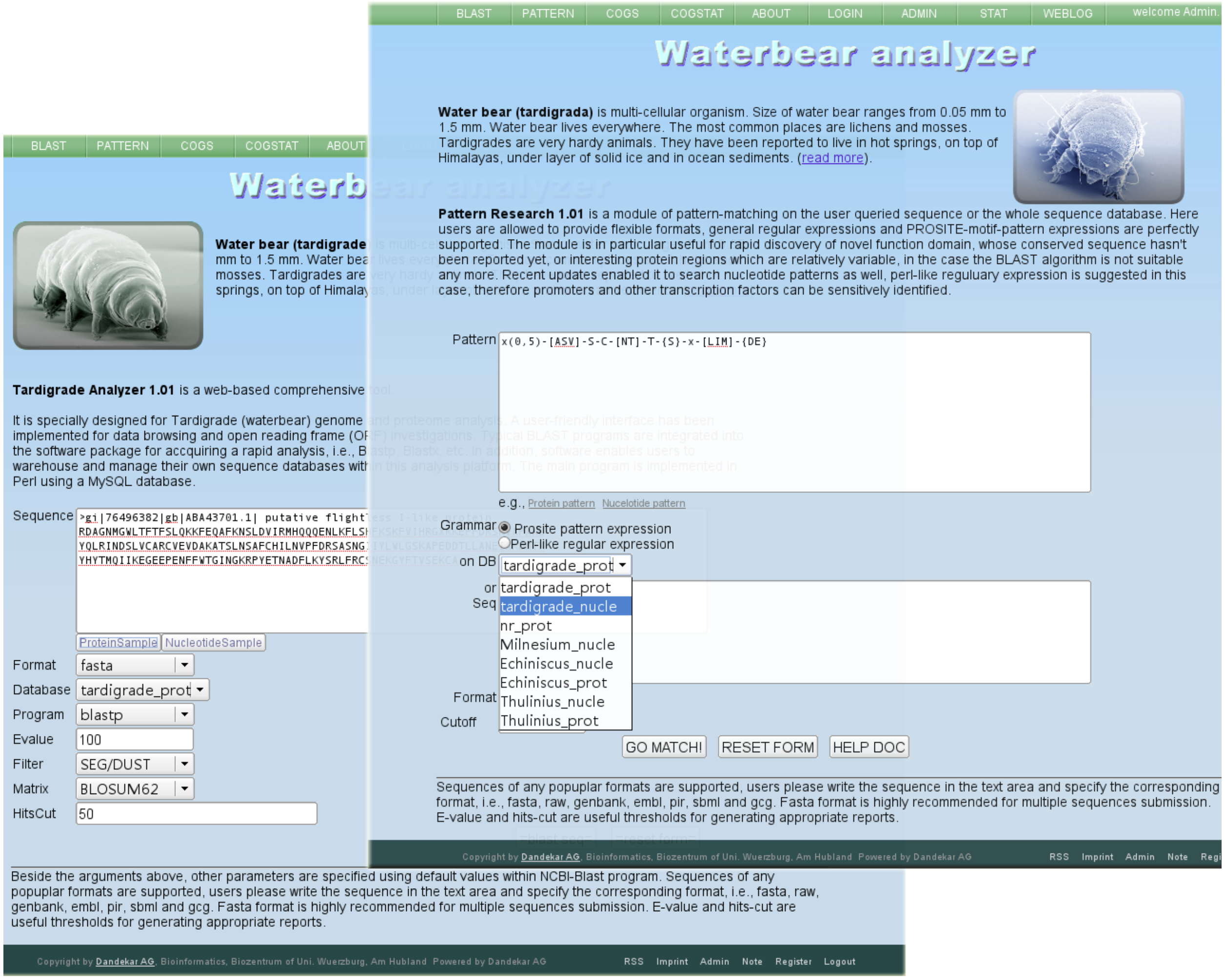

Figure S2: A snapshot of Glucocorticoid Response Element (GRE) pattern-searching results.

With the pattern-matching module, users can rapidly identify regulatory elements not only in RNA but also in DNA. This figure was generated using the Tardigrade analyzer, the specified pattern for the glucocorticoid receptor promotor element `AGAACAnnnTGTTCT` was searched against the nt database (all nucleotides of the nucleotid database as mirrored from NCBI). The complete report (hit, graphical localisation on the mRNA, sequence location and composition) can be readily obtained using the software.

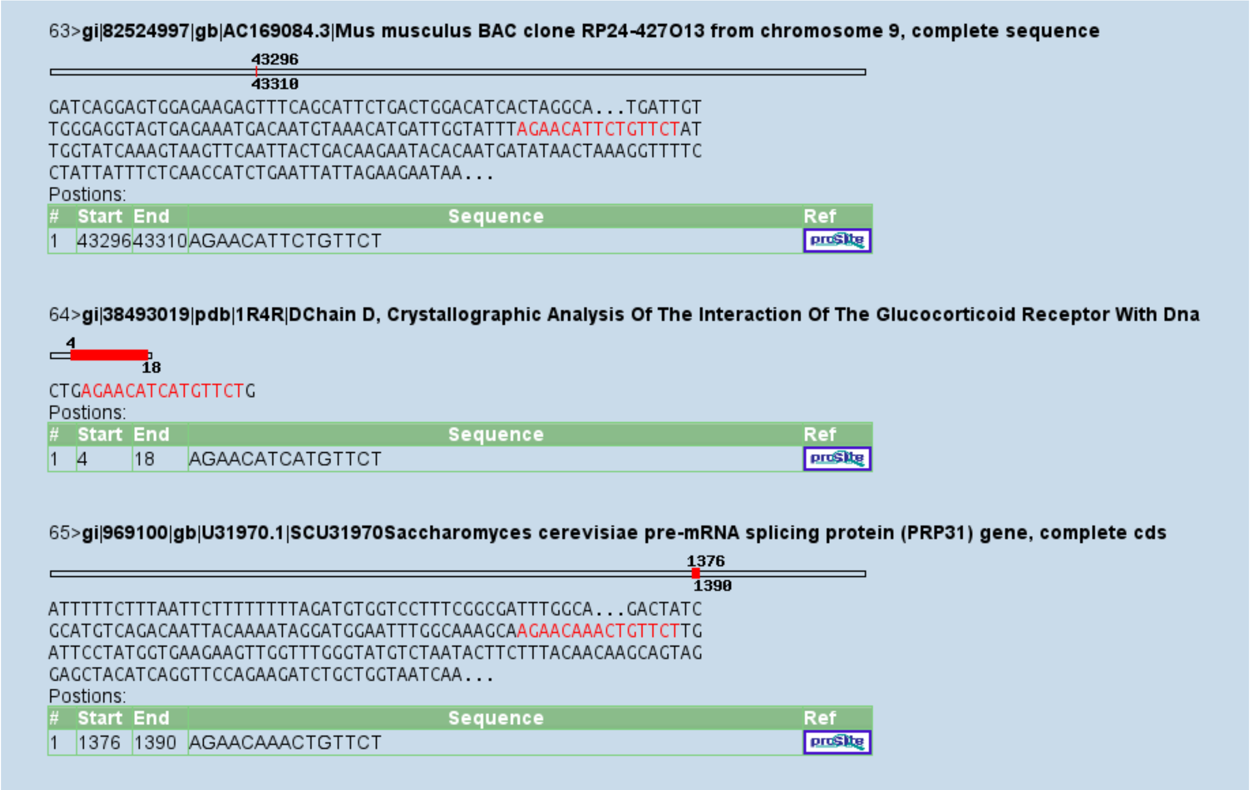

Figure S3: The user/data management function of the Tardigrade analyzer.

Among other options, users are allowed to create and operate their own database.

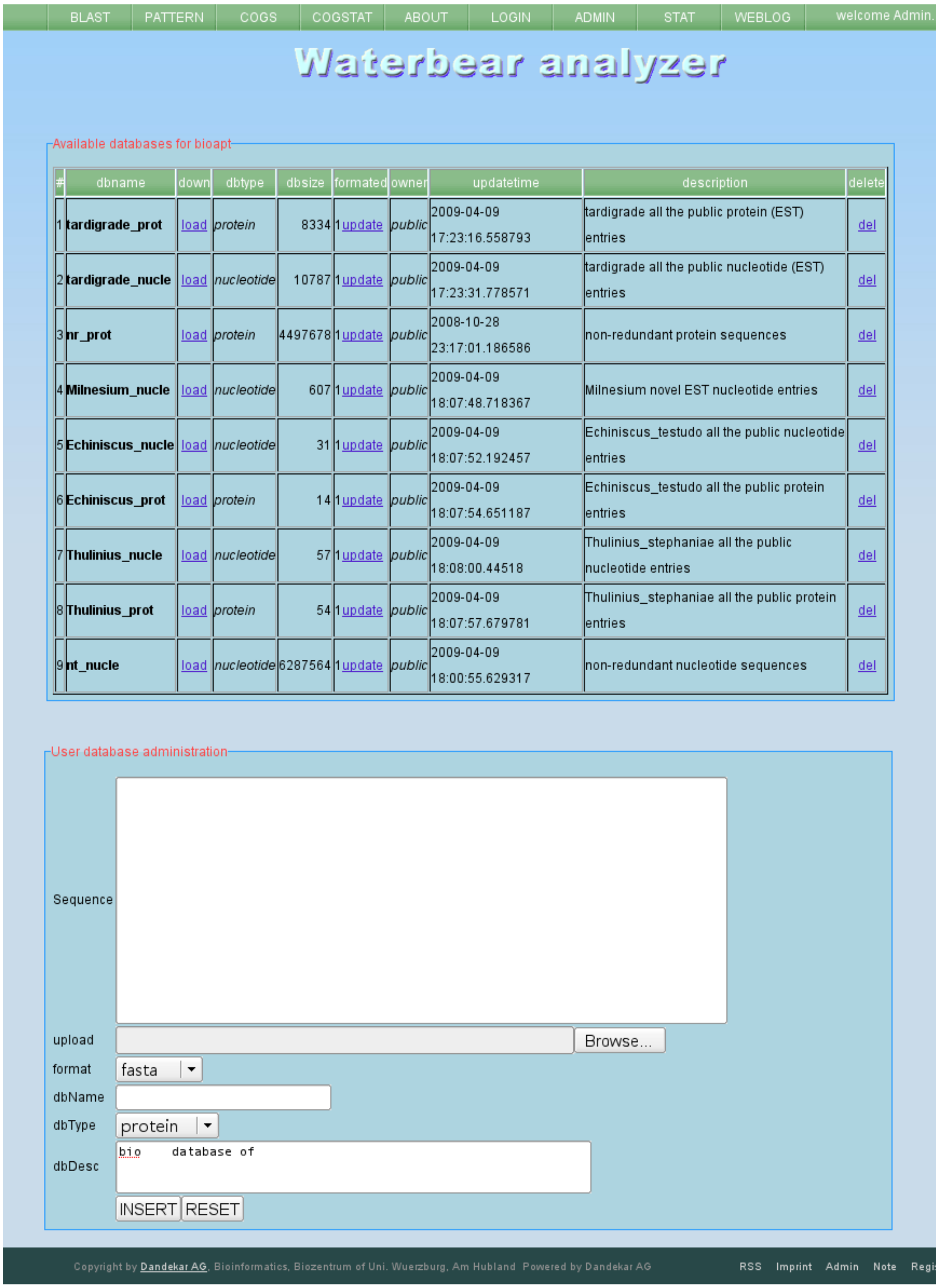

Figure S4: Report generated using the Tardigrade analyzer pattern-module.

A search for tardigrade polyadenylation sites is shown, the pattern applied was `AATTA{2,4}`. In the example given, the pattern was searched in all EST sequences of the Tardigrade-Analyzer, identified hits are shown in their position on the mRNA as well as sequence matched. Sequences are all given as DNA sequences (U replaced by a T).

256>**gi|37209346|gb|CD449046.1|CD449046|nottrimmed**

138 144 · 253 258

```
ATTTTCTTTTATAAAAAATTATAATTTAACTTTAAGGGTACTCGTGAGAGGCTTTTAAAA
AAATTTTTGAACTTTAAAACTTAAGTGTTTTGTGGGTAAGGATTTATTAAACTTTAAAGA
TTGTGTTCTTTTTCCCGAATTAAACTATTATTTTTATAAAAAGGATTTGAAACTGTAGAA
AAATTTTTTTAAAAAATATAAAAAGACAGTAAAACGCTATGAAGTTTAAGATATCTGGTT
ATTTAAGTTTCCAATTAAGTTGGAATTATGTTTATAAAGGTTTGTAAAGAAAAGTTTAGT
TTTCTTTTAAAATTTCAAAGAAAACCTTGCCATAATAAATTTTCCTTAATAGTTAATGGT
GCTTAGTATAGTTTTATAACT
```

Postions:

#	Start	End	Sequence	Ref
1	138	144	AATTAAA	prosite
2	253	258	AATTAA	prosite

257>**INTD2_A2_C06-M13-RP|nottrimmed**

212 217

```
ACAGGGATAGTCTTTATCATATTTTTTCAGTCatnnnctTTATCACAATT...AAATTAC
GATTagaACACAGAGCAAAGAGGCAAAAGCCAGCAAACGTCAGTAAATTAAGCGCTCTACAA
AATACGAGTAAAAAAGTGTTGCATCTCGC
```

Postions:

#	Start	End	Sequence	Ref
1	212	217	AATTAA	prosite

258>**gi|37210227|gb|CD449494.1|CD449494|nottrimmed**

138 144 · 253 258 · 417 424

```
ATTTTCTTTTATAAAAAATTATAATTTAACTTTAAGGGTACTCGTGAGAGGCTTTTAAAA
AAATTTTTGAACTTTAAAACTTAAGTGTTTTGTGGGTAAGGATTTATTAAACTTTAAAGA
TTGTGTTCTTTTTCCCGAATTAAACTATTATTTTTATAAAAAGGATTTGAAACTGTAGAA
AAATTTTTTTAAAAAATATAAAAAGACAGTAAAACGCTATGAAGTTTAAGATATCTGGTT
ATTTAAGTTTCCAATTAAGTTGGAATTATGTTTATAAAGGTTTGTAAAGAAAAGTTTAGT
TTTCTTTT...TATAGTTTTATAACTAAAAAAAATTTTTTTGTCTTCTTTTTTTCTTGTCTA
GAATTAAAATAATTGACAAAATATTTGTTGAAAAAATAATGATAAAATGAGTAGTTTGAA
CAAATTTGCATTAAAAAAATTTTACAAATAAATACAGCGACTGTNTAGCAAAAACATAGTT
TTTTGAATCTATTTAAAAAATTTGTTC
```

Postions:

#	Start	End	Sequence	Ref
1	138	144	AATTAAA	prosite
2	253	258	AATTAA	prosite
3	417	424	AATTAAAA	prosite

Figure S5: Protein cluster identification flowchart.

Detailed pictures are available on the Tardigrade analyzer WEB site (http://waterbear.bioapps.biozentrum.uni-wuerzburg.de/). Published tardigrade nucleotide sequences were collected (step 1) and clustered according to sequence similarity using the CLANS [8] algorithm (step 2). The largest protein clusters (all with at least 20 members) obtained were further functionally characterized by sequence analysis (10^{-3} E-value, both directions BLAST search including the re-check by reverse search, [9]; step 3).

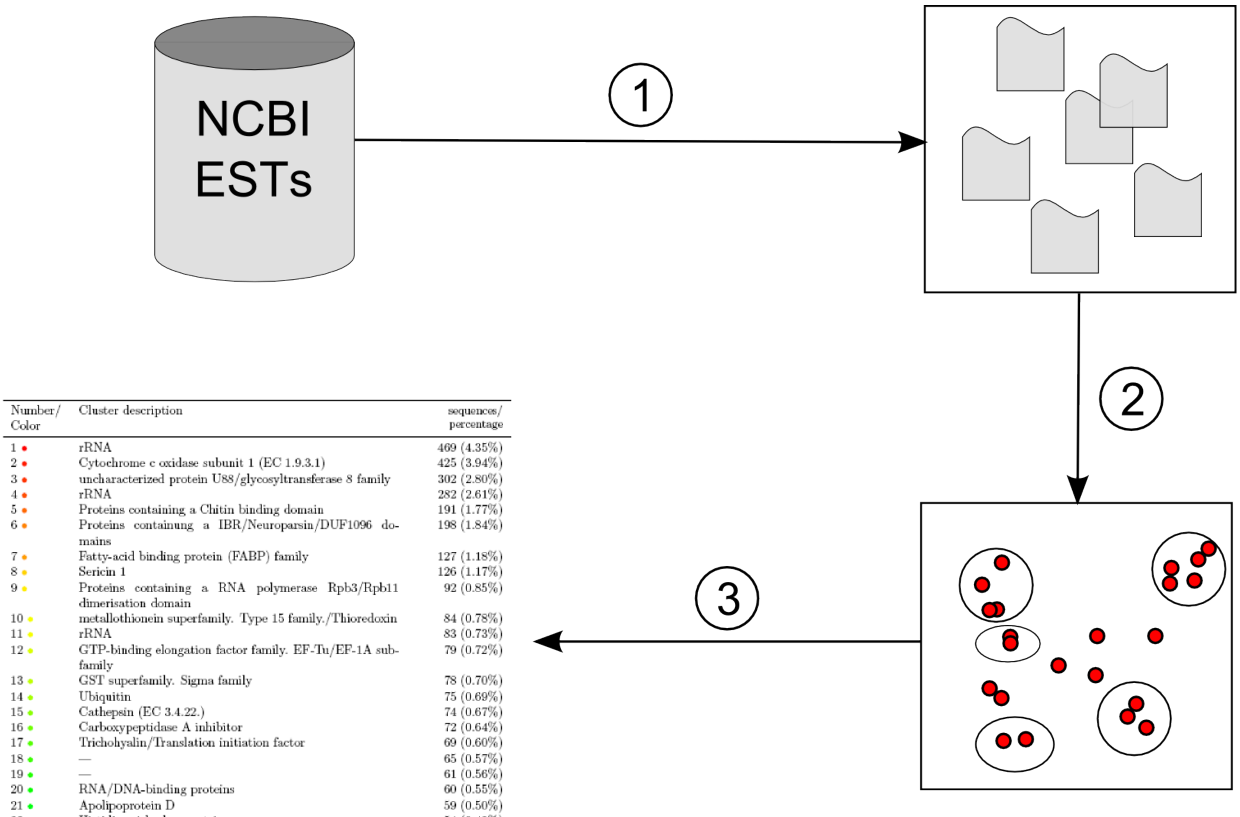

Number/ Color	Cluster description	sequences/ percentage
1 •	rRNA	469 (4.35%)
2 •	Cytochrome c oxidase subunit 1 (EC 1.9.3.1)	425 (3.94%)
3 •	uncharacterized protein U88/glycosyltransferase 8 family	302 (2.80%)
4 •	rRNA	282 (2.61%)
5 •	Proteins containing a Chitin binding domain	191 (1.77%)
6 •	Proteins containung a IBR/Neuroparsin/DUF1096 domains	198 (1.84%)
7 •	Fatty-acid binding protein (FABP) family	127 (1.18%)
8 •	Sericin 1	126 (1.17%)
9 •	Proteins containing a RNA polymerase Rpb3/Rpb11 dimerisation domain	92 (0.85%)
10 •	metallothionein superfamily. Type 15 family./Thioredoxin	84 (0.78%)
11 •	rRNA	83 (0.73%)
12 •	GTP-binding elongation factor family. EF-Tu/EF-1A subfamily	79 (0.72%)
13 •	GST superfamily. Sigma family	78 (0.70%)
14 •	Ubiquitin	75 (0.69%)
15 •	Cathepsin (EC 3.4.22.)	74 (0.67%)
16 •	Carboxypeptidase A inhibitor	72 (0.64%)
17 •	Trichohyalin/Translation initiation factor	69 (0.60%)
18 •	—	65 (0.57%)
19 •	—	61 (0.56%)
20 •	RNA/DNA-binding proteins	60 (0.55%)
21 •	Apolipoprotein D	59 (0.50%)
22 •	Histidine-rich glycoprotein	54 (0.49%)
23 •	small heat shock protein (HSP20) family	53 (0.47%)
24 •	Diapause-specific proteins	51 (0.44%)
25 •	cGMP-specific 3′, 5′-cyclic phosphodieesterase / Putative surface protein bspA-like	47 (0.42%)

Figure S6: Translation process flowchart.

Publicly available tardigrade nucleotide sequences were collected (step 1, Figure S6) and used for translation. To determine nucleotide sequences coding for known proteins, we did a BLASTX [1] search against the UniProtKB/SwissProt-, UniProtKB/TrEMBL- and NR-databases [2,3] (step 1) and in parallel a six frame translation using virtual ribosome [4] followed by a HMMER search [5] against the PFAM-database [6] (step 2). For all sequences resulting in a hit either in BLASTX or PFAM search (shown as lightblue connection lines) the corresponding ORFs were extracted (step 4). Sequences not identified via BLASTX were searched against the next more extensive database (connections shown in red). Sequences with no significant result either using BLASTX or using HMMER against PFAM were translated into six frames and all ORFs consisting of 100 or more amino acids were extracted. If no ORF had a length of 100 or more amino acids, we took the longest ORF (step 3). All sequences which seemed to be rRNA were identified using a BLASTN against a database of eukaryotic rRNAs [7]. These sequences were substracted from translated sequences (step 5). Finally sequences resulting in a hit in BLASTX and PFAM search (steps 1 and 2) were collapsed (step 6) and used in the next steps of the flowchart (Figure S6). Detailed pictures are available on the Tardigrade Analyzer WEB site.

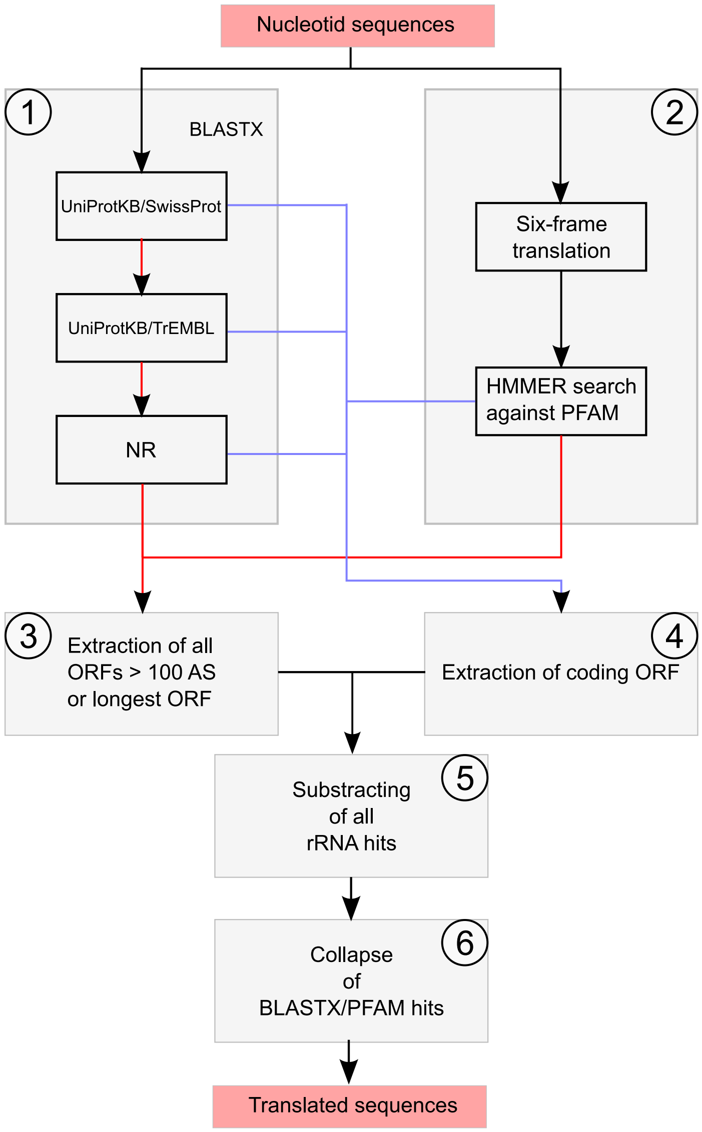
Nucleotid sequences
1
BLASTX
UniProtKB/SwissProt
UniProtKB/TrEMBL
NR
2
Six-frame translation
HMMER search against PFAM
3
Extraction of all ORFs > 100 AS or longest ORF
4
Extraction of coding ORF
5
Substracting of all rRNA hits
6
Collapse of BLASTX/PFAM hits
Translated sequences

Tables

Table S1: CLANS clusters of sequence similar proteins in published tardigrade sequences

Number/ color	Cluster description	Sequences/percentage[2]
1 ●	rRNA	469 (4.35%)
2 ●	Cytochrome c oxidase like (subunit 1, EC 1.9.3.1)	425 (3.94%)
3 ●	uncharacterized protein U88/glycosyltransferase 8 family	302 (2.80%)
4 ●	rRNA	282 (2.61%)
5 ●	Proteins containing a Chitin binding domain	191 (1.77%)
6 ●	Proteins containing an IBR/Neuroparsin/DUF1096 domain	189 (1.75%)
7 ●	Fatty-acid binding protein (FABP) family	127 (1.18%)
8 ●	TSP[1] remote homology to Sericin 1	126 (1.17%)
9 ●	Proteins containing a RNA polymerase Rpb3/Rpb11 dimerisation domain	92 (0.85%)
10 ●	Metallothionein superfamily (Type 15 family./Thioredoxin like)	84 (0.78%)
11 ●	rRNA	83 (0.73%)
12 ●	GTP-binding elongation factor family. EF-Tu/EF-1A subfamily	79 (0.72%)
13 ●	GST superfamily. Sigma family	78 (0.70%)
14 ●	Ubiquitin family	75 (0.69%)
15 ●	Cathepsin family (EC 3.4.22.-)	74 (0.67%)
16 ●	Carboxypeptidase A inhibitor like	72 (0.64%)
17 ●	Trichohyalin/Translation initiation factor like	69 (0.60%)
18 ●	TSP[1]	65 (0.57%)
19 ●	TSP[1]	61 (0.56%)
20 ●	RNA/DNA-binding proteins	60 (0.55%)
21 ●	Apolipoprotein D like	59 (0.50%)
22 ●	Histidine-rich glycoprotein like	54 (0.49%)
23 ●	small heat shock protein (HSP20) family	53 (0.47%)
24 ●	Diapause-specific proteins	51 (0.44%)
25 ●	cGMP-specific 3′, 5′-cyclic phosphodieesterase / Putative surface protein bspA-like	47 (0.42%)
26 ●	26S proteasome (BOP1NT (NUC169) domain or 26S proteasome subunit RPN7)	45 (0.42%)
27 ●	Sequestosome-1 like	45 (0.41%)
28 ●	small GTPase superfamily	44 (0.39%)
29 ●	Protein licA like	42 (0.38%)
30 ●	TSP[1]	41 (0.35%)
31 ●	Fatty-acid binding protein (FABP) family	38 (0.34%)
32 ●	Ribosomal protein L41 like	37 (0.33%)
33 ●	TSP[1]	36 (0.33%)
34 ●	Protein IWS1 homolog/Neuraminidase like	36 (0.32%)
35 ●	TSP[1]	34 (0.30%)
36 ●	Histidine-rich glycoprotein like	32 (0.30%)
37 ●	TSP[1]	32 (0.29%)

Continued on next page

Table S1 – continued from previous page

Number/ color	Cluster description	Sequences/percentage[2]
38 ●	LEA type 1 family proteins	31 (0.28%)
39 ●	Muscle LIM proteins	30 (0.27%)
40 ●	Entericidin EcnA/B family	29 (0.27%)
41 ●	Integrin, beta chain like	29 (0.27%)
42 ●	TSP[1]	29 (0.24%)
43 ●	ATP synthase subunit A like	26 (0.24%)
44 ●	Plasma membrane proteolipid 3 like	26 (0.23%)
45 ●	Actin family	25 (0.23%)
46 ●	Proteins containing a CD80-like C2-set immunoglobulin domain	29 (0.23%)
47 ●	Myosin light chain like proteins	25 (0.23%)
48 ●	Zinc metalloproteinase nas-Family (EC 3.4.24.21)	25 (0.22%)
49 ●	Protein Wnt-4 like	24 (0.21%)
50 ●	GABA(A) receptor-associated protein-like 1/2	23 (0.21%)
51 ●	TSP[1]	23 (0.20%)
52 ●	CUB and sushi domain-containing proteins	22 (0.19%)
53 ●	NADH dehydrogenase subunit 2 like (EC 1.6.5.3)	21 (0.19%)
54 ●	Eukaryotic translation initiation factor 4E-binding protein 2 like	21 (0.19%)
55 ●	TSP[1]	21 (0.19%)
56 ●	RNA polymerase II subunit B1 like (EC 2.7.7.6)	21 (0.19%)
57 ●	short chain dehydrogenase like	21 (0.19%)
58 ●	Niemann Pick type C2 protein homolog	20 (0.19%)

[1] Tardigrade specific proteins; [2] There was a total of 10787 sequences, percentage of this total is given in brackets.

Table S2: List of all new *Milnesium tardigradum* sequences.

dbEST_Id[1]	User_Id[2]	Accession[3]	Annotation[4]
62527332	INTD2_A1_A02	GE637185	no similarity
62527333	INTD2_A1_A04	GE637186	Receptor expression-enhancing protein 4
62527334	INTD2_A1_A06	GE637187	no similarity
62527335	INTD2_A1_A07	GE637188	no similarity
62527336	INTD2_A1_A08	GE637189	Vitellogenin-1
62527337	INTD2_A1_A10	GE637190	no similarity
62527338	INTD2_A1_A11	GE637191	no similarity
62527339	INTD2_A1_B01	GE637192	no similarity
62527340	INTD2_A1_B02	GE637193	no similarity
62527341	INTD2_A1_B03	GE637194	no similarity
62527342	INTD2_A1_B04	GE637195	40S ribosomal protein S21

Continued on next page

Table S2 – continued from previous page

dbEST_Id[1]	User_Id[2]	Accession[3]	Annotation[4]
62527343	INTD2_A1_B05	GE637196	60S ribosomal protein L10a
62527344	INTD2_A1_B06	GE637197	no similarity
62527345	INTD2_A1_B09	GE637198	no similarity
62527346	INTD2_A1_B11	GE637199	60S ribosomal protein L22-like 1
62527347	INTD2_A1_B12	GE637200	no similarity
62527348	INTD2_A1_C01	GE637201	no similarity
62527349	INTD2_A1_C02	GE637202	no similarity
62527350	INTD2_A1_C03	GE637203	Monoglyceride lipase
62527351	INTD2_A1_C04	GE637204	no similarity
62527352	INTD2_A1_C05	GE637205	Mitochondrial import inner membrane translocase subunit Tim23
62527353	INTD2_A1_C06	GE637206	no similarity
62527354	INTD2_A1_C07	GE637207	no similarity
62527355	INTD2_A1_C08	GE637208	no similarity
62527356	INTD2_A1_C09	GE637209	UPF0568 protein C14orf166 homolog
62527357	INTD2_A1_C10	GE637210	no similarity
62527358	INTD2_A1_D01	GE637211	no similarity
62527359	INTD2_A1_D02	GE637212	no similarity
62527360	INTD2_A1_D03	GE637213	no similarity
62527361	INTD2_A1_D05	GE637214	no similarity
62527362	INTD2_A1_D07	GE637215	no similarity
62527363	INTD2_A1_D09	GE637216	Threonyl-tRNA synthetase, cytoplasmic
62527364	INTD2_A1_D10	GE637217	no similarity
62527365	INTD2_A1_D12	GE637218	60S ribosomal protein L38
62527366	INTD2_A1_E03	GE637219	no similarity
62527367	INTD2_A1_E04	GE637220	40S ribosomal protein S21
62527368	INTD2_A1_E05	GE637221	no similarity
62527369	INTD2_A1_E06	GE637222	no similarity
62527370	INTD2_A1_E07	GE637223	B-cell receptor-associated protein 31
62527371	INTD2_A1_E08	GE637224	Golgi SNAP receptor complex member 1
62527372	INTD2_A1_E09	GE637225	no similarity
62527373	INTD2_A1_E11	GE637226	no similarity
62527374	INTD2_A1_E12	GE637227	no similarity
62527375	INTD2_A1_F01	GE637228	no similarity
62527376	INTD2_A1_F02	GE637229	no similarity
62527377	INTD2_A1_F03	GE637230	no similarity
62527378	INTD2_A1_F04	GE637231	dCTP pyrophosphatase 1
62527379	INTD2_A1_F07	GE637232	CDK-activating kinase assembly factor MAT1
62527380	INTD2_A1_F09	GE637233	no similarity
62527381	INTD2_A1_F10	GE637234	no similarity

Continued on next page

Table S2 – continued from previous page

dbEST_Id[1]	User_Id[2]	Accession[3]	Annotation[4]
62527382	INTD2_A1_F12	GE637235	no similarity
62527383	INTD2_A1_G01	GE637236	no similarity
62527384	INTD2_A1_G02	GE637237	Apolipoprotein D
62527385	INTD2_A1_G03	GE637238	no similarity
62527386	INTD2_A1_G04	GE637239	Cytochrome c oxidase subunit 3
62527387	INTD2_A1_G05	GE637240	no similarity
62527388	INTD2_A1_G06	GE637241	no similarity
62527389	INTD2_A1_G07	GE637242	no similarity
62527390	INTD2_A1_G08	GE637243	60S ribosomal protein L10a
62527391	INTD2_A1_G09	GE637244	no similarity
62527392	INTD2_A1_G10	GE637245	Probable pyruvate dehydrogenase E1 component subunit alpha, mitochondrial
62527393	INTD2_A1_G11	GE637246	no similarity
62527394	INTD2_A1_G12	GE637247	no similarity
62527395	INTD2_A1_H02	GE637248	no similarity
62527396	INTD2_A1_H04	GE637249	no similarity
62527397	INTD2_A1_H05	GE637250	no similarity
62527398	INTD2_A1_H06	GE637251	no similarity
62527399	INTD2_A1_H07	GE637252	no similarity
62527400	INTD2_A1_H08	GE637253	no similarity
62527401	INTD2_A1_H09	GE637254	no similarity
62527402	INTD2_A2_A01	GE637255	no similarity
62527403	INTD2_A2_A02	GE637256	no similarity
62527404	INTD2_A2_A04	GE637257	no similarity
62527405	INTD2_A2_A05	GE637258	no similarity
62527406	INTD2_A2_A06	GE637259	Elongation factor 1-alpha 1
62527407	INTD2_A2_A08	GE637260	Glutathione S-transferase
62527408	INTD2_A2_A09	GE637261	no similarity
62527409	INTD2_A2_A10	GE637262	no similarity
62527410	INTD2_A2_A11	GE637263	no similarity
62527411	INTD2_A2_B01	GE637264	no similarity
62527412	INTD2_A2_B02	GE637265	Mitogen-activated protein kinase scaffold protein 1-B
62527413	INTD2_A2_B04	GE637266	no similarity
62527414	INTD2_A2_B05	GE637267	no similarity
62527415	INTD2_A2_B06	GE637268	no similarity
62527416	INTD2_A2_B10	GE637269	Leech-derived tryptase inhibitor C
62527417	INTD2_A2_B11	GE637270	no similarity
62527418	INTD2_A2_B12	GE637271	no similarity
62527419	INTD2_A2_C01	GE637272	no similarity
62527420	INTD2_A2_C03	GE637273	no similarity
62527421	INTD2_A2_C04	GE637274	Cytochrome b
62527422	INTD2_A2_C05	GE637275	no similarity
62527423	INTD2_A2_C06	GE637276	no similarity

Continued on next page

Table S2 – continued from previous page

dbEST_Id[1]	User_Id[2]	Accession[3]	Annotation[4]
62527424	INTD2_A2_C08	GE637277	no similarity
62527425	INTD2_A2_C09	GE637278	no similarity
62527426	INTD2_A2_C10	GE637279	no similarity
62527427	INTD2_A2_C11	GE637280	no similarity
62527428	INTD2_A2_C12	GE637281	no similarity
62527429	INTD2_A2_D02	GE637282	no similarity
62527430	INTD2_A2_D04	GE637283	no similarity
62527431	INTD2_A2_D05	GE637284	no similarity
62527432	INTD2_A2_D06	GE637285	MMP37-like protein, mitochondrial
62527433	INTD2_A2_D07	GE637286	no similarity
62527434	INTD2_A2_D08	GE637287	no similarity
62527435	INTD2_A2_D09	GE637288	no similarity
62527436	INTD2_A2_D10	GE637289	Ovoinhibitor
62527437	INTD2_A2_E01	GE637290	Dolichyl-diphosphooligosaccharide–protein glycosyltransferase subunit 2
62527438	INTD2_A2_E02	GE637291	no similarity
62527439	INTD2_A2_E03	GE637292	no similarity
62527440	INTD2_A2_E04	GE637293	40S ribosomal protein S27
62527441	INTD2_A2_E06	GE637294	no similarity
62527442	INTD2_A2_E07	GE637295	no similarity
62527443	INTD2_A2_E08	GE637296	no similarity
62527444	INTD2_A2_E10	GE637297	no similarity
62527445	INTD2_A2_E11	GE637298	no similarity
62527446	INTD2_A2_E12	GE637299	no similarity
62527447	INTD2_A2_F02	GE637300	no similarity
62527448	INTD2_A2_F03	GE637301	no similarity
62527449	INTD2_A2_F04	GE637302	Isopentenyl-diphosphate Delta-isomerase 1
62527450	INTD2_A2_F05	GE637303	no similarity
62527451	INTD2_A2_F06	GE637304	no similarity
62527452	INTD2_A2_F07	GE637305	no similarity
62527453	INTD2_A2_F08	GE637306	no similarity
62527454	INTD2_A2_F09	GE637307	no similarity
62527455	INTD2_A2_F10	GE637308	no similarity
62527456	INTD2_A2_F11	GE637309	Prohibitin
62527457	INTD2_A2_F12	GE637310	no similarity
62527458	INTD2_A2_G01	GE637311	60S ribosomal protein L30
62527459	INTD2_A2_G03	GE637312	no similarity
62527460	INTD2_A2_G05	GE637313	Nicalin-1
62527461	INTD2_A2_G06	GE637314	no similarity
62527462	INTD2_A2_G07	GE637315	no similarity
62527463	INTD2_A2_G09	GE637316	Probable fatty acid-binding protein ENSP00000353650 homolog

Continued on next page

Table S2 – continued from previous page

dbEST_Id[1]	User_Id[2]	Accession[3]	Annotation[4]
62527464	INTD2_A2_G10	GE637317	no similarity
62527465	INTD2_A2_G11	GE637318	no similarity
62527466	INTD2_A2_H01	GE637319	no similarity
62527467	INTD2_A2_H02	GE637320	no similarity
62527468	INTD2_A2_H04	GE637321	no similarity
62527469	INTD2_A2_H05	GE637322	no similarity
62527470	INTD2_A2_H06	GE637323	26S protease regulatory subunit 4
62527471	INTD2_A2_H07	GE637324	no similarity
62527472	INTD2_A2_H08	GE637325	PiggyBac transposable element-derived protein 4
62527473	INTD2_A2_H09	GE637326	V-type proton ATPase subunit G
62527474	INTD2_A2_H10	GE637327	Histone-lysine N-methyltransferase MLL2
62527475	INTD2_A2_H12	GE637328	60S ribosomal protein L15
62527476	INTD2_B1_A01	GE637329	no similarity
62527477	INTD2_B1_A02	GE637330	no similarity
62527478	INTD2_B1_A03	GE637331	Lysosomal acid phosphatase
62527479	INTD2_B1_A04	GE637332	no similarity
62527480	INTD2_B1_A05	GE637333	no similarity
62527481	INTD2_B1_A06	GE637334	Translationally-controlled tumor protein homolog
62527482	INTD2_B1_A07	GE637335	no similarity
62527483	INTD2_B1_A08	GE637336	no similarity
62527484	INTD2_B1_A09	GE637337	no similarity
62527485	INTD2_B1_A10	GE637338	Glutathione S-transferase 1
62527486	INTD2_B1_A11	GE637339	Cathepsin B
62527487	INTD2_B1_A12	GE637340	Myotubularin-related protein 10
62527488	INTD2_B1_B01	GE637341	no similarity
62527489	INTD2_B1_B04	GE637342	Macrophage mannose receptor 1
62527490	INTD2_B1_B05	GE637343	ATP synthase subunit alpha
62527491	INTD2_B1_B06	GE637344	no similarity
62527492	INTD2_B1_B07	GE637345	Nuclear transcription factor Y subunit beta
62527493	INTD2_B1_B08	GE637346	no similarity
62527494	INTD2_B1_B09	GE637347	no similarity
62527495	INTD2_B1_B10	GE637348	no similarity
62527496	INTD2_B1_B11	GE637349	Transmembrane protein 14C
62527497	INTD2_B1_C02	GE637350	no similarity
62527498	INTD2_B1_C04	GE637351	no similarity
62527499	INTD2_B1_C05	GE637352	no similarity
62527500	INTD2_B1_C06	GE637353	no similarity
62527501	INTD2_B1_C07	GE637354	40S ribosomal protein S25
62527502	INTD2_B1_C08	GE637355	no similarity
62527503	INTD2_B1_C11	GE637356	Protein rogdi

Continued on next page

Table S2 – continued from previous page

dbEST_Id[1]	User_Id[2]	Accession[3]	Annotation[4]
62527504	INTD2_B1_C12	GE637357	Arginine kinase
62527505	INTD2_B1_D01	GE637358	no similarity
62527506	INTD2_B1_D03	GE637359	Serpin I2
62527507	INTD2_B1_D04	GE637360	NADH-ubiquinone oxidoreductase chain 4
62527508	INTD2_B1_D05	GE637361	no similarity
62527509	INTD2_B1_D06	GE637362	40S ribosomal protein S21
62527510	INTD2_B1_D07	GE637363	no similarity
62527511	INTD2_B1_D08	GE637364	no similarity
62527512	INTD2_B1_D09	GE637365	no similarity
62527513	INTD2_B1_D10	GE637366	no similarity
62527514	INTD2_B1_D12	GE637367	no similarity
62527515	INTD2_B1_E02	GE637368	no similarity
62527516	INTD2_B1_E04	GE637369	NADH dehydrogenase [ubiquinone] 1 alpha subcomplex subunit 13
62527517	INTD2_B1_E08	GE637370	no similarity
62527518	INTD2_B1_E10	GE637371	no similarity
62527519	INTD2_B1_E11	GE637372	60S ribosomal protein L30
62527520	INTD2_B1_E12	GE637373	Transforming growth factor-beta-induced protein ig-h3
62527521	INTD2_B1_F03	GE637374	NudC domain-containing protein 1
62527522	INTD2_B1_F04	GE637375	no similarity
62527523	INTD2_B1_F05	GE637376	Leech-derived tryptase inhibitor C
62527524	INTD2_B1_F06	GE637377	no similarity
62527525	INTD2_B1_F07	GE637378	Histone H4
62527526	INTD2_B1_F08	GE637379	UPF0368 protein Cxorf26 homolog
62527527	INTD2_B1_F09	GE637380	no similarity
62527528	INTD2_B1_F11	GE637381	28S ribosomal protein S30, mitochondrial
62527529	INTD2_B1_F12	GE637382	no similarity
62527530	INTD2_B1_G01	GE637383	Plasma alpha-L-fucosidase
62527531	INTD2_B1_G02	GE637384	no similarity
62527532	INTD2_B1_G03	GE637385	Endoplasmic reticulum protein ERp29
62527533	INTD2_B1_G04	GE637386	no similarity
62527534	INTD2_B1_G05	GE637387	no similarity
62527535	INTD2_B1_G06	GE637388	no similarity
62527536	INTD2_B1_G07	GE637389	no similarity
62527537	INTD2_B1_G08	GE637390	Glucan endo-1,3-beta-glucosidase A1
62527538	INTD2_B1_G09	GE637391	60S ribosomal protein L38
62527539	INTD2_B1_G10	GE637392	no similarity

Continued on next page

Table S2 – continued from previous page

dbEST_Id[1]	User_Id[2]	Accession[3]	Annotation[4]
62527540	INTD2_B1_G11	GE637393	no similarity
62527541	INTD2_B1_G12	GE637394	no similarity
62527542	INTD2_B1_H01	GE637395	no similarity
62527543	INTD2_B1_H02	GE637396	Actophorin
62527544	INTD2_B1_H03	GE637397	no similarity
62527545	INTD2_B1_H04	GE637398	U6 snRNA-associated Sm-like protein LSm3
62527546	INTD2_B1_H06	GE637399	40S ribosomal protein S15a
62527547	INTD2_B1_H08	GE637400	no similarity
62527548	INTD2_B1_H09	GE637401	no similarity
62527549	INTD2_B1_H10	GE637402	no similarity
62527550	INTD2_B1_H11	GE637403	no similarity
62527551	INTD2_B1_H12	GE637404	no similarity
62527552	INTD2_B2_A01	GE637405	Cytochrome c oxidase subunit 3
62527553	INTD2_B2_A03	GE637406	Cytochrome c oxidase subunit 6B
62527554	INTD2_B2_A05	GE637407	no similarity
62527555	INTD2_B2_A06	GE637408	Angiopoietin-related protein 7
62527556	INTD2_B2_A07	GE637409	no similarity
62527557	INTD2_B2_A08	GE637410	no similarity
62527558	INTD2_B2_A09	GE637411	Probable NADH dehydrogenase [ubiquinone] 1 alpha subcomplex subunit 12
62527559	INTD2_B2_A10	GE637412	Arginine kinase
62527560	INTD2_B2_A11	GE637413	AP-2 complex subunit sigma-1
62527561	INTD2_B2_B01	GE637414	no similarity
62527562	INTD2_B2_B03	GE637415	no similarity
62527563	INTD2_B2_B04	GE637416	G2/mitotic-specific cyclin-B
62527564	INTD2_B2_B08	GE637417	no similarity
62527565	INTD2_B2_B09	GE637418	no similarity
62527566	INTD2_B2_B12	GE637419	no similarity
62527567	INTD2_B2_C02	GE637420	Probable cationic amino acid transporter
62527568	INTD2_B2_C03	GE637421	no similarity
62527569	INTD2_B2_C04	GE637422	V-type proton ATPase subunit F
62527570	INTD2_B2_C05	GE637423	no similarity
62527571	INTD2_B2_C06	GE637424	no similarity
62527572	INTD2_B2_C08	GE637425	no similarity
62527573	INTD2_B2_C09	GE637426	Adrenodoxin, mitochondrial
62527574	INTD2_B2_C10	GE637427	no similarity
62527575	INTD2_B2_C11	GE637428	no similarity
62527576	INTD2_B2_D01	GE637429	Peptidyl-prolyl cis-trans isomerase
62527577	INTD2_B2_D03	GE637430	no similarity
62527578	INTD2_B2_D05	GE637431	no similarity
62527579	INTD2_B2_D07	GE637432	no similarity

Continued on next page

Table S2 – continued from previous page

dbEST_Id[1]	User_Id[2]	Accession[3]	Annotation[4]
62527580	INTD2_B2_D08	GE637433	60S ribosomal protein L27
62527581	INTD2_B2_D09	GE637434	no similarity
62527582	INTD2_B2_D12	GE637435	no similarity
62527583	INTD2_B2_E01	GE637436	Ovoinhibitor
62527584	INTD2_B2_E02	GE637437	Probable low affinity copper uptake protein 2
62527585	INTD2_B2_E03	GE637438	Cytochrome c oxidase subunit 3
62527586	INTD2_B2_E04	GE637439	no similarity
62527587	INTD2_B2_E05	GE637440	no similarity
62527588	INTD2_B2_E06	GE637441	no similarity
62527589	INTD2_B2_E07	GE637442	no similarity
62527590	INTD2_B2_E09	GE637443	Cathepsin L1
62527591	INTD2_B2_E10	GE637444	no similarity
62527592	INTD2_B2_E11	GE637445	no similarity
62527593	INTD2_B2_E12	GE637446	no similarity
62527594	INTD2_B2_F01	GE637447	no similarity
62527595	INTD2_B2_F02	GE637448	no similarity
62527596	INTD2_B2_F03	GE637449	no similarity
62527597	INTD2_B2_F04	GE637450	Glucan endo-1,3-beta-glucosidase A1
62527598	INTD2_B2_F05	GE637451	no similarity
62527599	INTD2_B2_F06	GE637452	ATP synthase subunit alpha, mitochondrial
62527600	INTD2_B2_F08	GE637453	no similarity
62527601	INTD2_B2_F09	GE637454	no similarity
62527602	INTD2_B2_F10	GE637455	NEDD8
62527603	INTD2_B2_F11	GE637456	no similarity
62527604	INTD2_B2_F12	GE637457	no similarity
62527605	INTD2_B2_G02	GE637458	no similarity
62527606	INTD2_B2_G03	GE637459	Syntaxin-18
62527607	INTD2_B2_G04	GE637460	no similarity
62527608	INTD2_B2_G06	GE637461	no similarity
62527609	INTD2_B2_G09	GE637462	Myosin-2 essential light chain
62527610	INTD2_B2_G10	GE637463	no similarity
62527611	INTD2_B2_G11	GE637464	no similarity
62527612	INTD2_B2_G12	GE637465	Transcription initiation factor TFIID subunit 7
62527613	INTD2_B2_H01	GE637466	Probable ATP-dependent RNA helicase DDX41
62527614	INTD2_B2_H02	GE637467	ATP synthase subunit a
62527615	INTD2_B2_H03	GE637468	no similarity
62527616	INTD2_B2_H04	GE637469	Glutathione S-transferase
62527617	INTD2_B2_H05	GE637470	no similarity
62527618	INTD2_B2_H06	GE637471	no similarity
62527619	INTD2_B2_H07	GE637472	no similarity
62527620	INTD2_B2_H08	GE637473	no similarity

Continued on next page

Table S2 – continued from previous page

dbEST_Id[1]	User_Id[2]	Accession[3]	Annotation[4]
62527621	INTD2_B2_H09	GE637474	Pre-mRNA-splicing factor cwc15
62527622	INTD2_B2_H10	GE637475	no similarity
62527623	INTD2_B2_H11	GE637476	no similarity
62527624	INTD2_B2_H12	GE637477	no similarity
62527625	ACTD1_A1_A02	GE637478	Small EDRK-rich factor 2
62527626	ACTD1_A1_A05	GE637479	no similarity
62527627	ACTD1_A1_A06	GE637480	no similarity
62527628	ACTD1_A1_A07	GE637481	no similarity
62527629	ACTD1_A1_A08	GE637482	40S ribosomal protein S28
62527630	ACTD1_A1_A09	GE637483	Cytochrome c oxidase subunit 7C, mitochondrial
62527631	ACTD1_A1_A10	GE637484	no similarity
62527632	ACTD1_A1_A11	GE637485	no similarity
62527633	ACTD1_A1_A12	GE637486	no similarity
62527634	ACTD1_A1_B01	GE637487	no similarity
62527635	ACTD1_A1_B02	GE637488	Transmembrane emp24 domain-containing protein 5
62527636	ACTD1_A1_B03	GE637489	no similarity
62527637	ACTD1_A1_B05	GE637490	no similarity
62527638	ACTD1_A1_B06	GE637491	no similarity
62527639	ACTD1_A1_B07	GE637492	40S ribosomal protein S7
62527640	ACTD1_A1_B08	GE637493	no similarity
62527641	ACTD1_A1_B09	GE637494	no similarity
62527642	ACTD1_A1_B10	GE637495	Protein FAM18B
62527643	ACTD1_A1_B11	GE637496	no similarity
62527644	ACTD1_A1_B12	GE637497	no similarity
62527645	ACTD1_A1_C01	GE637498	Atrial natriuretic peptide receptor B
62527646	ACTD1_A1_C03	GE637499	Probable signal peptidase complex subunit 2
62527647	ACTD1_A1_C05	GE637500	Comitin
62527648	ACTD1_A1_C07	GE637501	no similarity
62527649	ACTD1_A1_C08	GE637502	40S ribosomal protein S18
62527650	ACTD1_A1_C09	GE637503	no similarity
62527651	ACTD1_A1_C10	GE637504	60S ribosomal protein L35
62527652	ACTD1_A1_C11	GE637505	60S ribosomal protein L29
62527653	ACTD1_A1_D01	GE637506	30S ribosomal protein S6
62527654	ACTD1_A1_D02	GE637507	no similarity
62527655	ACTD1_A1_D03	GE637508	no similarity
62527656	ACTD1_A1_D04	GE637509	no similarity
62527657	ACTD1_A1_D06	GE637510	Cytochrome b
62527658	ACTD1_A1_D07	GE637511	no similarity
62527659	ACTD1_A1_D08	GE637512	Cytohesin-1
62527660	ACTD1_A1_D09	GE637513	no similarity
62527661	ACTD1_A1_D10	GE637514	no similarity
62527662	ACTD1_A1_E01	GE637515	no similarity

Continued on next page

Table S2 – continued from previous page

dbEST_Id[1]	User_Id[2]	Accession[3]	Annotation[4]
62527663	ACTD1_A1_E02	GE637516	no similarity
62527664	ACTD1_A1_E03	GE637517	Platelet-activating factor acetyl-hydrolase IB subunit beta
62527665	ACTD1_A1_E04	GE637518	no similarity
62527666	ACTD1_A1_E07	GE637519	no similarity
62527667	ACTD1_A1_E08	GE637520	ATP synthase subunit d, mitochondrial
62527668	ACTD1_A1_E09	GE637521	Transcription factor E2F4
62527669	ACTD1_A1_E10	GE637522	no similarity
62527670	ACTD1_A1_E11	GE637523	no similarity
62527671	ACTD1_A1_E12	GE637524	no similarity
62527672	ACTD1_A1_F01	GE637525	40S ribosomal protein S3a
62527673	ACTD1_A1_F02	GE637526	DNA-directed RNA polymerases I, II, and III subunit RPABC2
62527674	ACTD1_A1_F04	GE637527	Cytochrome c oxidase subunit 3
62527675	ACTD1_A1_F05	GE637528	no similarity
62527676	ACTD1_A1_F06	GE637529	no similarity
62527677	ACTD1_A1_F07	GE637530	Myophilin
62527678	ACTD1_A1_F08	GE637531	60S ribosomal protein L22
62527679	ACTD1_A1_F09	GE637532	no similarity
62527680	ACTD1_A1_F10	GE637533	no similarity
62527681	ACTD1_A1_F11	GE637534	Vitellogenin-1
62527682	ACTD1_A1_F12	GE637535	no similarity
62527683	ACTD1_A1_G01	GE637536	no similarity
62527684	ACTD1_A1_G02	GE637537	no similarity
62527685	ACTD1_A1_G03	GE637538	no similarity
62527686	ACTD1_A1_G06	GE637539	Cytochrome b
62527687	ACTD1_A1_G07	GE637540	no similarity
62527688	ACTD1_A1_G08	GE637541	no similarity
62527689	ACTD1_A1_G09	GE637542	no similarity
62527690	ACTD1_A1_G10	GE637543	no similarity
62527691	ACTD1_A1_G11	GE637544	no similarity
62527692	ACTD1_A1_G12	GE637545	Malectin
62527693	ACTD1_A1_H01	GE637546	Actin-related protein 2/3 complex subunit 4
62527694	ACTD1_A1_H02	GE637547	no similarity
62527695	ACTD1_A1_H03	GE637548	no similarity
62527696	ACTD1_A1_H05	GE637549	no similarity
62527697	ACTD1_A1_H06	GE637550	COMM domain-containing protein 3
62527698	ACTD1_A1_H07	GE637551	Myosin essential light chain, striated adductor muscle
62527699	ACTD1_A1_H08	GE637552	Elongation factor 1-alpha
62527700	ACTD1_A1_H09	GE637553	no similarity
62527701	ACTD1_A1_H10	GE637554	Eukaryotic translation initiation factor 3 subunit C

Continued on next page

Table S2 – continued from previous page

dbEST_Id[1]	User_Id[2]	Accession[3]	Annotation[4]
62527702	ACTD1_A2_A01	GE637555	no similarity
62527703	ACTD1_A2_A02	GE637556	no similarity
62527704	ACTD1_A2_A03	GE637557	TIP41-like protein
62527705	ACTD1_A2_A04	GE637558	40S ribosomal protein S23
62527706	ACTD1_A2_A06	GE637559	40S ribosomal protein S15
62527707	ACTD1_A2_A07	GE637560	no similarity
62527708	ACTD1_A2_A09	GE637561	no similarity
62527709	ACTD1_A2_A10	GE637562	no similarity
62527710	ACTD1_A2_A11	GE637563	Serine/threonine-protein phosphatase 2B catalytic subunit 3
62527711	ACTD1_A2_A12	GE637564	no similarity
62527712	ACTD1_A2_B01	GE637565	no similarity
62527713	ACTD1_A2_B02	GE637566	40S ribosomal protein S15
62527714	ACTD1_A2_B03	GE637567	Ovoinhibitor
62527715	ACTD1_A2_B04	GE637568	Acid ceramidase
62527716	ACTD1_A2_B05	GE637569	Histone H4
62527717	ACTD1_A2_B06	GE637570	no similarity
62527718	ACTD1_A2_B07	GE637571	no similarity
62527719	ACTD1_A2_B08	GE637572	no similarity
62527720	ACTD1_A2_B09	GE637573	no similarity
62527721	ACTD1_A2_B10	GE637574	no similarity
62527722	ACTD1_A2_B11	GE637575	Extracellular peptidase inhibitor
62527723	ACTD1_A2_B12	GE637576	no similarity
62527724	ACTD1_A2_C01	GE637577	Fatty acid-binding protein 2
62527725	ACTD1_A2_C02	GE637578	no similarity
62527726	ACTD1_A2_C03	GE637579	RING-box protein 1
62527727	ACTD1_A2_C04	GE637580	60S ribosomal protein L10a
62527728	ACTD1_A2_C05	GE637581	no similarity
62527729	ACTD1_A2_C06	GE637582	no similarity
62527730	ACTD1_A2_C08	GE637583	no similarity
62527731	ACTD1_A2_C09	GE637584	Glycyl-tRNA synthetase 1
62527732	ACTD1_A2_C10	GE637585	Mannose-P-dolichol utilization defect 1 protein
62527733	ACTD1_A2_C11	GE637586	no similarity
62527734	ACTD1_A2_C12	GE637587	no similarity
62527735	ACTD1_A2_D02	GE637588	no similarity
62527736	ACTD1_A2_D03	GE637589	Ubiquitin
62527737	ACTD1_A2_D04	GE637590	no similarity
62527738	ACTD1_A2_D05	GE637591	no similarity
62527739	ACTD1_A2_D06	GE637592	Comitin
62527740	ACTD1_A2_D07	GE637593	40S ribosomal protein S21
62527741	ACTD1_A2_D08	GE637594	no similarity
62527742	ACTD1_A2_D10	GE637595	Thioredoxin-1
62527743	ACTD1_A2_D11	GE637596	no similarity

Continued on next page

Table S2 – continued from previous page

dbEST_Id[1]	User_Id[2]	Accession[3]	Annotation[4]
62527744	ACTD1_A2_D12	GE637597	Meiotic recombination protein SPO11
62527745	ACTD1_A2_E01	GE637598	no similarity
62527746	ACTD1_A2_E02	GE637599	no similarity
62527747	ACTD1_A2_E05	GE637600	40S ribosomal protein S29
62527748	ACTD1_A2_E06	GE637601	no similarity
62527749	ACTD1_A2_E08	GE637602	Transaldolase
62527750	ACTD1_A2_E09	GE637603	no similarity
62527751	ACTD1_A2_E10	GE637604	no similarity
62527752	ACTD1_A2_E11	GE637605	Leech-derived tryptase inhibitor C
62527753	ACTD1_A2_F01	GE637606	60S ribosomal protein L35
62527754	ACTD1_A2_F02	GE637607	no similarity
62527755	ACTD1_A2_F03	GE637608	no similarity
62527756	ACTD1_A2_F04	GE637609	no similarity
62527757	ACTD1_A2_F06	GE637610	no similarity
62527758	ACTD1_A2_F07	GE637611	Transportin-2
62527759	ACTD1_A2_F09	GE637612	no similarity
62527760	ACTD1_A2_F10	GE637613	Cofilin
62527761	ACTD1_A2_F11	GE637614	Platelet-activating factor acetyl-hydrolase IB subunit beta
62527762	ACTD1_A2_F12	GE637615	Succinyl-CoA ligase [GDP-forming] subunit beta, mito-chondrial
62527763	ACTD1_A2_G01	GE637616	Beta-1,4-galactosyltransferase 1
62527764	ACTD1_A2_G02	GE637617	no similarity
62527765	ACTD1_A2_G03	GE637618	no similarity
62527766	ACTD1_A2_G04	GE637619	Ubiquitin
62527767	ACTD1_A2_G05	GE637620	no similarity
62527768	ACTD1_A2_G07	GE637621	no similarity
62527769	ACTD1_A2_G08	GE637622	no similarity
62527770	ACTD1_A2_G09	GE637623	no similarity
62527771	ACTD1_A2_G10	GE637624	no similarity
62527772	ACTD1_A2_G11	GE637625	no similarity
62527773	ACTD1_A2_G12	GE637626	no similarity
62527774	ACTD1_A2_H01	GE637627	Eukaryotic translation initiation factor 5B
62527775	ACTD1_A2_H02	GE637628	no similarity
62527776	ACTD1_A2_H04	GE637629	no similarity
62527777	ACTD1_A2_H05	GE637630	no similarity
62527778	ACTD1_A2_H06	GE637631	no similarity
62527779	ACTD1_A2_H07	GE637632	no similarity
62527780	ACTD1_A2_H08	GE637633	no similarity
62527781	ACTD1_A2_H09	GE637634	no similarity
62527782	ACTD1_A2_H10	GE637635	no similarity
62527783	ACTD1_A2_H11	GE637636	no similarity

Continued on next page

Table S2 – continued from previous page

dbEST_Id[1]	User_Id[2]	Accession[3]	Annotation[4]
62527784	ACTD1_A2_H12	GE637637	Eukaryotic translation initiation factor 5A
62527785	ACTD1_B1_A01	GE637638	no similarity
62527786	ACTD1_B1_A02	GE637639	no similarity
62527787	ACTD1_B1_A03	GE637640	no similarity
62527788	ACTD1_B1_A04	GE637641	60S ribosomal protein L23
62527789	ACTD1_B1_A05	GE637642	no similarity
62527790	ACTD1_B1_A07	GE637643	40S ribosomal protein S15
62527791	ACTD1_B1_A08	GE637644	no similarity
62527792	ACTD1_B1_A09	GE637645	no similarity
62527793	ACTD1_B1_A11	GE637646	no similarity
62527794	ACTD1_B1_B01	GE637647	no similarity
62527795	ACTD1_B1_B02	GE637648	Peptidyl-prolyl cis-trans isomerase, mitochondrial
62527796	ACTD1_B1_B03	GE637649	no similarity
62527797	ACTD1_B1_B04	GE637650	Cytochrome b
62527798	ACTD1_B1_B05	GE637651	Signal recognition particle 9 kDa protein
62527799	ACTD1_B1_B06	GE637652	no similarity
62527800	ACTD1_B1_B07	GE637653	40S ribosomal protein S12
62527801	ACTD1_B1_B08	GE637654	Transmembrane protein 147
62527802	ACTD1_B1_B09	GE637655	Cdc42 homolog
62527803	ACTD1_B1_B10	GE637656	Superoxide dismutase [Cu-Zn], chloroplastic
62527804	ACTD1_B1_B11	GE637657	no similarity
62527805	ACTD1_B1_B12	GE637658	Actin-related protein 2/3 complex subunit 1A
62527806	ACTD1_B1_C01	GE637659	no similarity
62527807	ACTD1_B1_C02	GE637660	no similarity
62527808	ACTD1_B1_C03	GE637661	no similarity
62527809	ACTD1_B1_C04	GE637662	Deoxyhypusine synthase
62527810	ACTD1_B1_C06	GE637663	Cathepsin Z
62527811	ACTD1_B1_C07	GE637664	no similarity
62527812	ACTD1_B1_C08	GE637665	no similarity
62527813	ACTD1_B1_C09	GE637666	no similarity
62527814	ACTD1_B1_C10	GE637667	Troponin C
62527815	ACTD1_B1_C11	GE637668	no similarity
62527816	ACTD1_B1_D03	GE637669	no similarity
62527817	ACTD1_B1_D04	GE637670	28S ribosomal protein S36, mitochondrial
62527818	ACTD1_B1_D05	GE637671	no similarity
62527819	ACTD1_B1_D07	GE637672	Alpha-aspartyl dipeptidase
62527820	ACTD1_B1_D09	GE637673	no similarity
62527821	ACTD1_B1_D10	GE637674	no similarity
62527822	ACTD1_B1_D11	GE637675	no similarity
62527823	ACTD1_B1_D12	GE637676	no similarity

Continued on next page

Table S2 – continued from previous page

dbEST_Id[1]	User_Id[2]	Accession[3]	Annotation[4]
62527824	ACTD1_B1_E04	GE637677	no similarity
62527825	ACTD1_B1_E05	GE637678	Mitochondrial import inner membrane translocase subunit Tim9
62527826	ACTD1_B1_E06	GE637679	no similarity
62527827	ACTD1_B1_E07	GE637680	Phosphatidylethanolamine-binding protein homolog F40A3.3
62527828	ACTD1_B1_E08	GE637681	no similarity
62527829	ACTD1_B1_E10	GE637682	Thaumatin-like protein 2
62527830	ACTD1_B1_E11	GE637683	no similarity
62527831	ACTD1_B1_E12	GE637684	no similarity
62527832	ACTD1_B1_F01	GE637685	NEDD8
62527833	ACTD1_B1_F04	GE637686	no similarity
62527834	ACTD1_B1_F05	GE637687	no similarity
62527835	ACTD1_B1_F06	GE637688	no similarity
62527836	ACTD1_B1_F07	GE637689	Dynactin subunit 2
62527837	ACTD1_B1_F08	GE637690	Ubiquitin
62527838	ACTD1_B1_F09	GE637691	Isochorismatase domain-containing protein 1
62527839	ACTD1_B1_F11	GE637692	no similarity
62527840	ACTD1_B1_F12	GE637693	Translocation protein SEC63 homolog
62527841	ACTD1_B1_G02	GE637694	no similarity
62527842	ACTD1_B1_G03	GE637695	no similarity
62527843	ACTD1_B1_G04	GE637696	no similarity
62527844	ACTD1_B1_G05	GE637697	no similarity
62527845	ACTD1_B1_G06	GE637698	no similarity
62527846	ACTD1_B1_G07	GE637699	no similarity
62527847	ACTD1_B1_G08	GE637700	no similarity
62527848	ACTD1_B1_G09	GE637701	DNA-directed RNA polymerase II subunit RPB11
62527849	ACTD1_B1_G10	GE637702	no similarity
62527850	ACTD1_B1_G11	GE637703	no similarity
62527851	ACTD1_B1_G12	GE637704	no similarity
62527852	ACTD1_B1_H02	GE637705	no similarity
62527853	ACTD1_B1_H03	GE637706	no similarity
62527854	ACTD1_B1_H04	GE637707	no similarity
62527855	ACTD1_B1_H06	GE637708	no similarity
62527856	ACTD1_B1_H07	GE637709	no similarity
62527857	ACTD1_B1_H08	GE637710	no similarity
62527858	ACTD1_B1_H09	GE637711	no similarity
62527859	ACTD1_B1_H10	GE637712	Vitellogenin-1
62527860	ACTD1_B1_H11	GE637713	Peroxiredoxin-5, mitochondrial
62527861	ACTD1_B1_H12	GE637714	60S ribosomal protein L30
62527862	ACTD1_B2_A02	GE637715	no similarity

Continued on next page

Table S2 – continued from previous page

dbEST_Id[1]	User_Id[2]	Accession[3]	Annotation[4]
62527863	ACTD1_B2_A03	GE637716	40S ribosomal protein S10
62527864	ACTD1_B2_A04	GE637717	no similarity
62527865	ACTD1_B2_A05	GE637718	no similarity
62527866	ACTD1_B2_A06	GE637719	no similarity
62527867	ACTD1_B2_A07	GE637720	60S ribosomal protein L23
62527868	ACTD1_B2_A08	GE637721	no similarity
62527869	ACTD1_B2_A09	GE637722	no similarity
62527870	ACTD1_B2_A10	GE637723	NADH dehydrogenase [ubiquinone] 1 beta subcomplex subunit 7
62527871	ACTD1_B2_A11	GE637724	no similarity
62527872	ACTD1_B2_A12	GE637725	Aquaporin-9
62527873	ACTD1_B2_B02	GE637726	Probable 28S ribosomal protein S6, mitochondrial
62527874	ACTD1_B2_B03	GE637727	Vacuolar ATPase assembly integral membrane protein VMA21
62527875	ACTD1_B2_B04	GE637728	Uncharacterized protein C4orf34 homolog
62527876	ACTD1_B2_B05	GE637729	Peptidyl-prolyl cis-trans isomerase
62527877	ACTD1_B2_B06	GE637730	Transmembrane protein 50A
62527878	ACTD1_B2_B07	GE637731	no similarity
62527879	ACTD1_B2_C01	GE637732	no similarity
62527880	ACTD1_B2_C02	GE637733	no similarity
62527881	ACTD1_B2_C03	GE637734	no similarity
62527882	ACTD1_B2_C05	GE637735	no similarity
62527883	ACTD1_B2_C06	GE637736	Mitochondrial 2-oxoglutarate/malate carrier protein
62527884	ACTD1_B2_C07	GE637737	Cytochrome c oxidase subunit 5A, mitochondrial
62527885	ACTD1_B2_C09	GE637738	no similarity
62527886	ACTD1_B2_C10	GE637739	no similarity
62527887	ACTD1_B2_C11	GE637740	no similarity
62527888	ACTD1_B2_C12	GE637741	Ankyrin repeat domain-containing protein 17
62527889	ACTD1_B2_D02	GE637742	Phosphoenolpyruvate carboxykinase [GTP]
62527890	ACTD1_B2_D03	GE637743	no similarity
62527891	ACTD1_B2_D05	GE637744	Chitosanase
62527892	ACTD1_B2_D06	GE637745	no similarity
62527893	ACTD1_B2_D08	GE637746	no similarity
62527894	ACTD1_B2_D10	GE637747	no similarity
62527895	ACTD1_B2_D11	GE637748	no similarity
62527896	ACTD1_B2_D12	GE637749	Heat-stable enterotoxin receptor
62527897	ACTD1_B2_E02	GE637750	no similarity

Continued on next page

Table S2 – continued from previous page

dbEST_Id[1]	User_Id[2]	Accession[3]	Annotation[4]
62527898	ACTD1_B2_E03	GE637751	NADH-ubiquinone oxidoreductase chain 4
62527899	ACTD1_B2_E04	GE637752	no similarity
62527900	ACTD1_B2_E05	GE637753	Ribosomal protein S6 kinase alpha-1
62527901	ACTD1_B2_E06	GE637754	40S ribosomal protein S15a
62527902	ACTD1_B2_E07	GE637755	no similarity
62527903	ACTD1_B2_E08	GE637756	Protein FRA10AC1 homolog
62527904	ACTD1_B2_E10	GE637757	Ubiquitin
62527905	ACTD1_B2_E11	GE637758	no similarity
62527906	ACTD1_B2_E12	GE637759	no similarity
62527907	ACTD1_B2_F01	GE637760	40S ribosomal protein S25
62527908	ACTD1_B2_F03	GE637761	no similarity
62527909	ACTD1_B2_F04	GE637762	ATP synthase subunit a
62527910	ACTD1_B2_F05	GE637763	no similarity
62527911	ACTD1_B2_F06	GE637764	no similarity
62527912	ACTD1_B2_F07	GE637765	no similarity
62527913	ACTD1_B2_F08	GE637766	no similarity
62527914	ACTD1_B2_F09	GE637767	ATP synthase subunit a
62527915	ACTD1_B2_F11	GE637768	Ribosome biogenesis protein NSA2 homolog
62527916	ACTD1_B2_F12	GE637769	no similarity
62527917	ACTD1_B2_G01	GE637770	no similarity
62527918	ACTD1_B2_G02	GE637771	no similarity
62527919	ACTD1_B2_G03	GE637772	no similarity
62527920	ACTD1_B2_G06	GE637773	no similarity
62527921	ACTD1_B2_G07	GE637774	no similarity
62527922	ACTD1_B2_G08	GE637775	no similarity
62527923	ACTD1_B2_G09	GE637776	no similarity
62527924	ACTD1_B2_G10	GE637777	no similarity
62527925	ACTD1_B2_G11	GE637778	no similarity
62527926	ACTD1_B2_G12	GE637779	no similarity
62527927	ACTD1_B2_H01	GE637780	Histone H4
62527928	ACTD1_B2_H02	GE637781	no similarity
62527929	ACTD1_B2_H04	GE637782	General stress protein 69
62527930	ACTD1_B2_H05	GE637783	no similarity
62527931	ACTD1_B2_H06	GE637784	Probable tyrosyl-tRNA synthetase, mitochondrial
62527932	ACTD1_B2_H07	GE637785	no similarity
62527933	ACTD1_B2_H08	GE637786	no similarity
62527934	ACTD1_B2_H09	GE637787	no similarity
62527935	ACTD1_B2_H10	GE637788	Glutathione S-transferase
62527936	ACTD1_B2_H11	GE637789	60S ribosomal protein L7
62527937	ACTD1_B2_B09	GE637790	Probable fatty acid-binding protein ENSP00000353650 homolog
62565092	ACTD1_B2_B10	GE653116	no similarity

[1] ID number given by dbEST; [2] Name of the sequence; [3] Accession number of the corresponding sequence

given by Genbank; [4] Annotation of the sequences
